# Bio-friendly multi-stimuli responsive α-CD polymer-gated mesoporous carbon nanoherbicides for enhanced paraquat delivery

**DOI:** 10.1016/j.jare.2024.12.005

**Published:** 2024-12-12

**Authors:** Jiangtao Dong, Guoquan Wang, Xiaona Li, Aohui Han, Wanpeng Zhang, Yuhang Yue, Yue Yang, Yishan Wang, Bowen Yuan, Jiahui Wang, Yuhui Peng, Runqiang Liu, Si Chen, Xuezhong Du

**Affiliations:** a College of Resources and Environment, Henan Institute of Science and Technology, Xinxiang 453003, Henan Province, PR China; b Henan Engineering Research Center of Green Pesticide Creation & Intelligent Pesticide Residue Sensor Detection, Henan Institute of Science and Technology, Xinxiang 453003, Henan Province, PR China; c School of Food Science, Henan Institute of Science and Technology, Xinxiang 453003, Henan Province, PR China; d Key Laboratory of Mesoscopic Chemistry (Ministry of Education), School of Chemistry and Chemical Engineering, Nanjing University, Nanjing 210023, PR China

**Keywords:** Paraquat, α–CD polymer gatekeepers, Mesoporous carbon nanocarriers, Multi-stimuli responsive controlled release, Efficient weed control, Non-target biosafety

## Abstract

**Introduction:**

Weeds seriously affect crop yield in global agricultural production. Paraquat (PQ), as one of low cost and highly effective herbicide, is forbidden or severely restricted in production and sales owing to its lethal toxicity to humans. Creating an efficient and bio-friendly PQ formulation is crucial to facilitate the open use of PQ in world’s agriculture.

**Objectives:**

This study aims to construct one intelligent and bio-friendly mesoporous carbon nanoparticles (MCN) nanoherbicides coated with α-CD polymer (CDP) gatekeepers.

**Methods:**

MCN was prepared through the low-concentration hydrothermal way, calcined and carbonized. PEG stalks were immobilized on MCN surface by amidation reaction. The PQ was trapped in the MCN pores via physical diffusion adsorption and the robust π–π effects between electron-deficient PQ and electron-rich MCN. CDP gatekeepers were fastened via host–guest effects between the chamber of α-CD units and PEG stalks.

**Results:**

The PQ-loaded MCN-PEG@CDP nanoherbicides integrated with multi-stimuli responses to amylase, elevated temperature under sunlight, and competitors at leaf interface to control the PQ release for efficient weed control, while appeared low PQ leakage under the simulated human gastric or intestinal conditions, low cytotoxicity to human normal cells *in vitro*, and high mouse survival rate *in vivo*. Even through the nanoherbicides inevitably contact with water or intake by beneficial insects, they appear good biosafety on zebrafish (*D. rerio*) and honeybees (*Apis mellifera L*.).

**Conclusion:**

The as-prepared nanoherbicides have high herbicidal efficacy and low risks to non-target species, and could promote the open use of PQ in agriculture.

## Highlights

• Bio-friendly paraquat mesoporous carbon nanoherbicides were created.
• The capping of α–CD polymer was hitched via host–guest effect.
• The nanoherbicides possessed controllable herbicidal activity in response to multidimensional stimuli.
• The nanoherbicides showed good biosafety on model mouse, zebrafish, and honeybees.

## Graphical abstract

**Figure:**
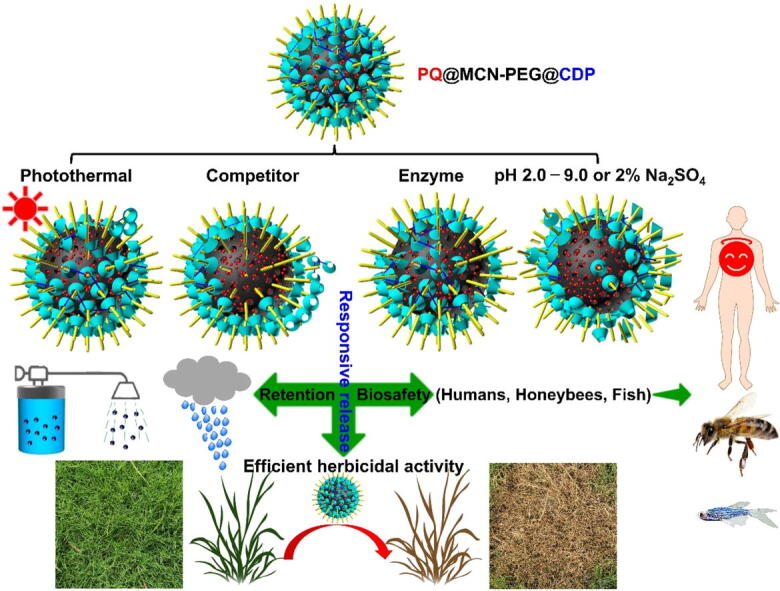

## Introduction

Weeds refer to all plants that grow on production sites that are adverse or harmful to human activities. Paraquat (PQ, 1,1′-dimethyl-4,4′-bipyridinium dichloride), as one of low cost, non-selective, and highly effective herbicide, is very popular in global agriculture and gardening [1], [2], [3]. PQ can become deactivated in the soil and invalid for plant roots, perennial rhizomes, and seeds hidden in the soil [2], [4]. At present, there is no effective antidote to combat the ingestion of PQ based on the lethal toxicity of PQ to humans, thus commercial PQ formulations are forbidden or severely restricted in production and sales in some countries [2], [3].

In recent decades, researchers have devoted to create one bio-friendly and user-safe PQ formulations, while still maintain the effective weed control. Syngenta created a PQ formulation (named as Inteon), containing three sorts of additives: alginate used to reduce gastric absorption, a laxative used to eliminate PQ from the intestine, and an emetic used to dislodge PQ from the stomach [2], [5]. However, the expected effects on weed control and human safety were not achieved after administration of interon. Baltazar et al. prepared a safe PQ formulation containing the known antidote lysine acetylsalicylate, which presented significantly decreases mammalian toxicity against PQ-triggered lung damage and effective weed control [3]. Peng et al. reported a new PQ surrogate named dienediamine (the “reduced” form of PQ), which appeared nontoxicity *in vivo*/*vitro* experiments at molar contents (same as the PQ's absolute mortal dosage), and potent weed control resulting from the resultful transformation to PQ under sunlight [6]. Besides, for the purpose of avoiding gastrointestinal absorption, several detoxification strategies against PQ intake for humans were designed, such as the administration of gastric lavage or mineral adsorbents (e.g., activated charcoal, attapulgite, diatomite, or bentonite), which all have limited efficacy for PQ formulations in practical application [7], [8], [9].

Nanopesticides are selected by IUPAC as one of the ten chemical innovations to change the world in 2019 [10], which maybe solve the challenge against the lethal toxicity of PQ absorption to humans based on the intelligent controlled release, low dosage, and enhanced bioavailability for target crop plants [11], [12], [13], [14], [15], [16], [17], [18]. To whittle the high morbidity and mortality and improve the utilization efficacy of PQ, different kinds of PQ nanoherbicides have been designed to achieve intelligent controlled release and biosafety *in vitro*/*vivo*. Wang et al. constructed an effective UV-light stimuli-responsive PQ supramolecular vesicles based on the ternary macrocyclic self-assembly via host–guest effects, which consists of the technical PQ, cucurbit[8]uril, and azobenzene derivant [19]. However, under the physiological conditions, owing to the immanent instability of the vesicle structure, PQ trapped in the internal cavities of vesicles will inevitably generate early release. Furthermore, they prepared one hyaluronic acid (HA)-coated ternary macrocyclic nanoparticles (NPs) based on the self-assembly via host–guest effects among the PQ-linked polylactic acid NPs, cucurbit[8]uril, and *trans*-azobenzene-functionalized HA, which appeared availably biosafety by the decoration of HA film, as demonstrated in zebrafish and mouse models, and retained good herbicidal activity [20]. As previously reported, we prepared chitosan gated porous carbon nanoherbicides for the controlled release of encapsulated PQ in response to acidic substrate and high temperature, which appeared low damage to human normal cells *in vitro*, high survival rate of mice *in vivo*, and effective weed control [21]. In addition, we also prepared one carboxymethyl cellulose-Ca^II^ coordinated networks gated metal–organic framework MIL-101(Fe^III^) nanoherbicides, which integrated with five stimuli-responsive (GSH, PO_4_^3–^, acidic/alkaline pH, or EDTA), and showed good herbicidal efficacy via the controlled release of encapsulated PQ and good biosafety for honeybees [22].

Mesoporous carbon nanoparticles (MCN) have been broadly studied in biotechnology and nanomedicine attributing to the good biocompatibility, high specific surface areas, large pore volumes, and customizable uniform pore structures [23]. As drug/pesticide carriers, MCN possesses immanent hydrophobicity, low toxicity, and scarce immunogenic property [24], [25], [26], which could serve as an ideal delivery platform to transport cargos to the target sites in nanomedicines and nanopesticides. Moreover, based on the broadband light absorption, MCN could as the photothermal transducers under NIR radiation, converting NIR light into thermal energy to drive the controlled release of loaded cargos [27], [28], [29]. Cyclodextrins (CD) are kind of cyclic oligosaccharides formed from glucopyranose units linked by 1,4-glycosidic bonds, forming an internally hydrophobic and externally hydrophilic truncated pyramid structure. The hydrophobic inner chamber of CD can accommodate different kind of hydrophobic segments or hydrophobic molecules [30], [31], [32], [33]. The poly(ethylene glycol)s (PEG) chain fits well into the chamber of α–CD (diameter of the chamber = 4.5 Å) and could shape inclusion complexes with α–CD via host–guest effects [34], [35], [36]. Compared to parent cyclodextrin (CD) monomer, studies have shown that the cyclodextrin polymer (CDP) has good water solubility, which is ca. about 8.6 times higher than original CD [37].

**Scheme 1:**
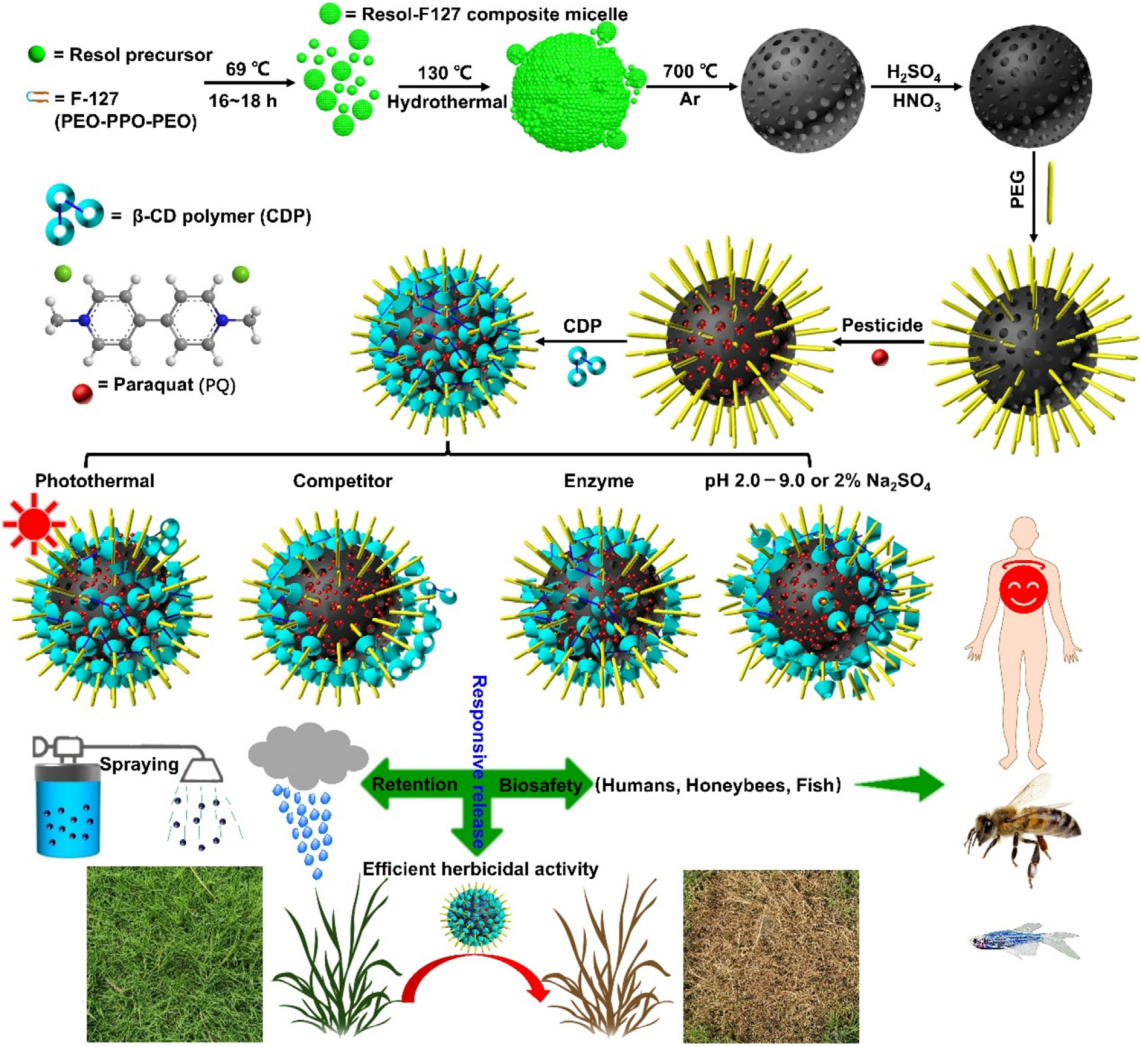
Illustration of the creation of cyclodextrin polymer (CDP)-gated MCN nanoherbicides loaded with paraquat (PQ), multi-stimuli responsive controlled release of loaded technical PQ (sunlight, competitor, and enzyme), efficient control of outdoor weeds, and biosafety for humans, honeybees and zebrafish.

The point of this work is to create one bio-friendly and multi-stimuli responsive PQ nanoherbicides. Herein, MCN was prepared through the low-concentration hydrothermal way, calcined and carbonized at 700 °C in N_2_, and further carboxylated on the surface to obtain MCN-COOH [23], [38]. NH_2_-PEG chains can be immobilized on the surface of MCN-COOH through amidation reaction [39]. The technical PQ could be trapped in the pores of MCN mainly based on physical diffusion adsorption, and even the robust π–π effects between PQ (electron deficiency) and MCN (electron enrichment). Subsequently, the highly water-soluble and low-cost CDP were used as gatekeepers to coat the PQ-loaded MCN-PEG on account of host–guest effects between the chamber of α–CD units in CDP and PEG stalks on the MCN-COOH surface to obtain CDP-gated MCN (PQ@MCN-PEG@CDP) nanoherbicides (Scheme 1). PEG chains modified MCN (MCN-PEG) could not only improve biocompatibility and dispersibility of MCN in water to reduce or avoid the addition of additives, but serve as stalks to construct gatekeepers with CDP based on the host–guest effects. The coating of CDP on the MCN surface can not only prevent PQ-encapsulated in pores of MCN early leakage, but facilitate the adhesion and retention of MCN on hydrophobic leaf surface based on the boost of wettability and spreading.

Upon the deposition of the PQ@MCN-PEG@CDP nanoherbicides on the plant leaf surface, the hydrophobic molecules of epicuticular wax cyclic and aliphatic compounds [e.g. decylic acid (DA) or ursolic acid (UA)] [40], [41] could also form inclusion complexes with the chamber of α–CD units in CDP. Both UA and DA as leaf surface competitors could cause CDP to dethread from the PEG stalks, opening the gatekeepers and releasing the encapsulated PQ due to the host–guest competitive binding. α–Amylase can hydrolyze the 1,4-glycosidic bonds of CD [42], [43], [44], [45], causing CDP to decompose and release the encapsulated PQ. Besides, MCN has a high photothermal conversion efficiency (PCE) based on the strong absorption of NIR because the total solar irradiation energy contains 52 % NIR irradiation energy [46], which could weaken the host–guest effects of the nanovalves and even the π–π effects between the PQ (electron deficiency) and the MCN (electron enrichment), leading to the sunlight responsive controlled release of encapsulated PQ. Thus, the as-prepared nanoherbicides integrated with multi-stimuli responses to amylase, competitors at leaf interface, and elevated temperature under sunlight for the controlled release of trapped PQ (Scheme 1). Moreover, it is completely different from the clinical treatment of PQ poisoning where activated charcoal is used as PQ adsorbent to impede PQ gastrointestinal absorption [5], [7], [8], [9]. The release of encapsulated PQ could be inhibited even in the acidic gastric matrix (pH as low as 2.0), or the laxative (e.g. 2 % Na_2_SO_4_ aqueous solution), let alone in the neutral or alkaline intestine matrix (pH 7.0–9.0) (Scheme 1), which indicates that it is safe for users and can avoid PQ leakage in the matrix of gastrointestinal tract from the root against the challenge of gastrointestinal PQ absorption. The as-prepared nanoherbicides appeared good weed control, low cytotoxicity to human normal cells *in vitro*, and high mouse survival rate *in vivo*. In addition, compared with the technical PQ, the as-prepared nanoherbicides could avoid the off-target loss based on good spreading and retention on target plant leaf surface, and reduce ecological hazards based on good biosafety on zebrafish and honeybees. It is shown the PQ@MCN-PEG@CDP nanoherbicides could be developed a promising bio-friendly PQ formulation in future agriculture.

## Methods

### Synthesis, carboxylation, and PEG functionalization of MCN

Small-size MCN was synthesized via the low-concentration hydrothermal way [23]. Phenol (0.6 g), 37 % formaldehyde (2.1 mL) aqueous solution, and 0.1 M NaOH (15 mL) aqueous solution were mixed and stirred at 70 °C for 0.5 h to obtain low-molecular-weight phenolic resols, then Pluronic F127 (0.96 g) dissolved in 15 mL of water was added. The mixed solution was stirred at 69 °C for 3 h, then 50 mL of water was added to dilute the mixed solution. After 17 h, the reaction was terminated. Then 17.7 mL of solution was placed in stainless steel reactor, and followed by addition of 56 mL of water, heating at 130 °C for 24 h. The product was collected, and washed by water for 4–6 times. The carbonization of the obtained product was administered at 700 °C in an N_2_ atmosphere for about 3 h. The Pluronic F127 templates were removed during this process, thus the initial MCN was synthesized. The as-synthesized MCN was placed into concentrated H_2_SO_4_ and HNO_3_ (v/v, 3:1) for carboxylation (MCN-COOH) via ultrasound. The NH_2_-PEG stalks functionalized MCN (MCN-C = O-NH-PEG, abbreviated as MCN-PEG) was synthesized via the amidation reaction (formed by condensation reaction between anhydride and amine). One hundred milligrams of MCN were dispersed in 20 mL of anhydrous dichloromethane solution, EDC (0.16 g, 0.8 mmol) and NHS (0.10 g, 0.8 mmol) were added to activate the carboxyl group, and 20 μL of triethylamine as catalyst was added and reacted for 12 h. NH_2_-PEG-COOH (100 mg, MW = 2000 Da) dissolved in DMSO was added to the solution. The reaction was continued for 24 h under N_2_ atmosphere in the dark. The product was collected by centrifugation and dialyzed with a dialysis bag (MW cutoff = 7000). The MCN-PEG was obtained via vacuum drying at 60 °C.

### Synthesis of α–CD polymer (CDP) gatekeepers

The α–CD polymer (CDP) gatekeepers were synthesized through the linking of epichlorohydrin (EP) among α–CD units in basic solution according to the modified method in the paper (Fig. S1) [47], [48]. The ^1^H NMR, ^13^C NMR, and FTIR spectra of CDP are afforded in Fig. S2–S4. Experimental details are in Supporting Information.

### Preparation of PQ nanoherbicides (PQ@MCN-PEG@CDP)

The capping of CDP gatekeepers was fastened via host–guest effects between the chamber of α–CD units in CDP and PEG stalks on the MCN surface. MCN-PEG (40 mg) was dispersed in 6 mM technical PQ aqueous solution, and stirred for 24 h in the dark. PQ could be loaded in the pore of MCN-PEG via physical diffusion and π-π effects (An interaction that frequently occurs between aromatic rings, typically between two molecules that are relatively electron rich or electron deficient, is a non-covalent bond interaction and equally important as hydrogen bonding). The PQ loaded MCN-PEG was retrieved via centrifugation, and placed in 10 mg mL^−1^ of CDP (10 mL) aqueous solution, and stirred for 24 h. The products were collected and washed by water 3–4 times to remove the superfluous CDP. The PQ@MCN-PEG@CDP nanoherbicides were obtained by freeze-dried way.

### Controlled release of the technical PQ from PQ@MCN-PEG@CDP nanoherbicides

The as-prepared PQ@MCN-PEG@CDP nanoherbicides of equal weight were added to various aqueous solution with Tween-80 (0.1 %). The water solution at various pH, different temperatures, with 2 % Na_2_SO_4_, different molar concentrations of UA and DA at pH 7.2, only with different fractions of critical micelle concentration (CMC) of AOT and Tween-80, and in presence of α–amylase were used for the responsive release of the technical PQ. A fraction of the target aqueous solution was extracted at the given time to test the released PQ via HPLC, and the fresh aqueous media of equal volume was added timely.

### Deposition and retention of PQ@MCN-PEG@CDP

The aqueous dispersion of PQ@MCN-PEG@CDP or FITC@MCN-PEG@CDP (0.5 mg mL^−1^, 10 mL) was sprayed uniformly on the surface of weed leaves at a 60° bevel to the horizontal, respectively. The treated weed leaves (before and after washing) were surveyed by SEM or confocal laser scanning microscope. The excitation wavelength of FITC was 488 nm. Each experiment was repeated three times.

### The safety evaluation of PQ@MCN-PEG@CDP *in vitro* / *in vivo*

1) The human normal hepatic cells (LO-2) were collected to survey the cell damage of technical PQ, MCN-PEG@CDP, and PQ@MCN-PEG@CDP using MTT assay (*n* = 6). 2) Female C57BL/6 mice (6 weeks old) were intragastrically administrated with technical PQ at 20 mg kg^−1^, MCN-PEG@CDP at 290 mg kg^−1^, and PQ@MCN-PEG@CDP at 290 mg kg^−1^ (same as the PQ dose of 7 mg kg^−1^) on the first day, respectively, and the survival rates were recorded over a 14-day period (*n* = 6). Meanwhile, the body weights of the mice in different groups were test (*n* = 6). At the end of experiment, all mice were sacrificed, and the blood were collected for hematology analysis (*n* = 3) and biochemical assay (*n* = 3), including hepatic damage biomarkers (ALT and AST) and renal function biomarkers (UA and BUN). The heart, liver, spleen, lung, and kidney were performed for H&E staining (*n* = 3).

### Herbicidal efficacy of PQ@MCN-PEG@CDP nanoherbicides

The technical PQ (0.2 g L^–1^), MCN-PEG@CDP (3.0 g L^–1^), and PQ@MCN-PEG@CDP (1.0 and 3.0 g L^–1^, respectively same as 0.068 and 0.2 g L^–1^ of the technical PQ) were dispersed in water solutions with Tween-80 (0.1 %), respectively. The samples (*n* = 3) were sprayed against outdoor weeds (*Cynodon dactylon*) in an area of 0.16 m^2^ (0.4 × 0.4 m). Eight milliliter of each sample was administered for 3 consecutive days.

### Safety of PQ@MCN-PEG@CDP nanoherbicides on non-target species zebrafish and honeybees

In order to evaluate the effects of PQ@MCN-PEG@CDP nanoherbicides on aquatic ecosystem, zebrafish (*D. rerio*) were used as experimental model. The semi-static method was used to evaluate the acute toxicity of zebrafish. They were acclimated under experimental conditions for 7 days before the experiment. zebrafish were randomly selected with a total length of 1.5 ∼ 2.5 cm, normal body color, healthy and lively, and an average body weight of 0.200 g. The experimental water was filtered aerated tap water, and the temperature of 25 ± 1°C, pH adjusted to 6.5 by 1.0 M HCl, hardness of 114 mg/L, light/dark ratio of 14/10 h. Stop feeding 24 h before formal experiment. The volume of the test solution was 500 mL. A series of concentrations of PQ@MCN-PEG@CDP nanoherbicides (respectively same as 25, 50, 75, 100, and 125 μg mL^−1^ of technical PQ) were set up in the experiment with 8 zebrafish per group (*n* = 8 × 3). The test solution was replaced with fresh solution at 24, 48, 72, and 96 h after the start of the test. The pH, water temperature, and dissolved oxygen content were measured simultaneously. The poisoning symptoms and death of zebrafish were observed and recorded at each designed point. The criteria for determining death are to touch the fish's tail with a glass rod. Each experiment was repeated three times.

The ingestion method was used to evaluate the acute oral toxicity of honeybees (*Apis mellifera L*.). Two hours of starvation treatment before the experiment. Fifty honeybees per group (*n* = 50 × 3) were randomly selected and incubated in a feeding box, and fed with 50 % sucrose solution containing the dosages of 1470 and 2945 μg mL^−1^ PQ@MCN-PEG@CDP nanoherbicides (respectively same as 100 and 200 μg mL^−1^ of technical PQ). The numbers of deaths after 24 and 48 h were recorded to calculate lethal rate of honeybees. Each experiment was repeated three times.

### Data analysis.

All treatments were run with at least 3 replicates. The median effective concentration (*EC*_50_) for the survival curves was calculated by probit regression analysis. Comparisons of the leaf retention amount, fluorescence image intensity, cell viability, liver and renal function biomarkers, herbicidal efficacy, survival rates, and biosecurity results were analyzed using one-way analysis of variance (ANOVA) and Duncan’s multiple range test. *P < 0.05 and ^**^P < 0.01 were considered as significant and highly significant, respectively. The statistical analyses were performed using Data Processing Station SPSS 22.0 (IBM, USA). The charts and graphs of data were presented by OriginPro 8.5 (OriginLab, USA). Graphed data are shown as means ± standard errors.

## Results and discussion

### Characterizations and preparation of PQ@MCN-PEG@CDP nanoherbicides

**Fig. 1:**
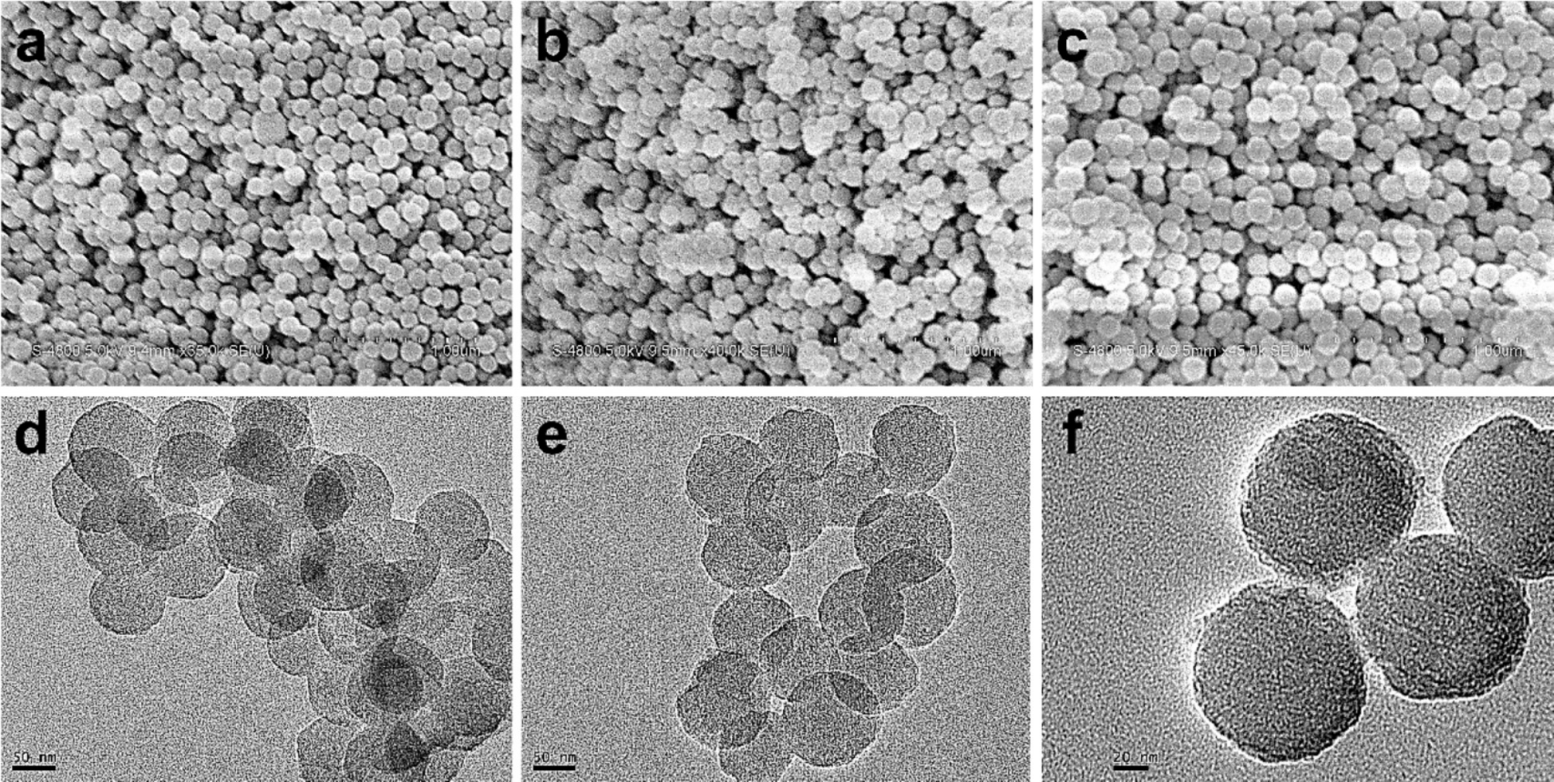
SEM photographs of (a) MCN, (b) MCN-PEG, and (c) MCN-PEG@CDP and counterpart images of TEM (d–f).

Small-size MCN with uniform spherical morphology was prepared via the low-concentration hydrothermal way based on the previous reported method [23], using the phenolic resin as the carbon feeding and Pluronic F127 as the template framework, and followed by calcination and carbonization at 700 °C under N_2_ atmosphere. During this process, F127 scaffolds were also eliminated. The carboxyl (–COOH) groups were created on the surface of MCN via ultrasonic treatment in HNO_3_ and H_2_SO_4_ (v/v, 1:3) mixed solution. The photographs of the carboxylated MCN appear regular spherical shape with a medial size of 85.9 nm by scanning electron microscope (SEM) and transmission electron microscope (TEM), respectively (Fig. 1a, d). The morphologies of MCN functionalized by PEG stalks (indicated as MCN-PEG) via amidation reaction remained almost unchanged by the images of SEM and TEM, respectively (Fig. 1b, e). The MCN-PEG@CDP was constructed by the coating of CDP gatekeepers via host–guest effects between PEG stalks on the MCN surface and the chamber of α–CD units in CDP. The SEM and TEM photographs of MCN-PEG@CDP appear a layer of rough aureole on the surface resulting from the coating of CDP, with a medial size of 96.7 nm (Fig. 1c, f).

**Fig. 2:**
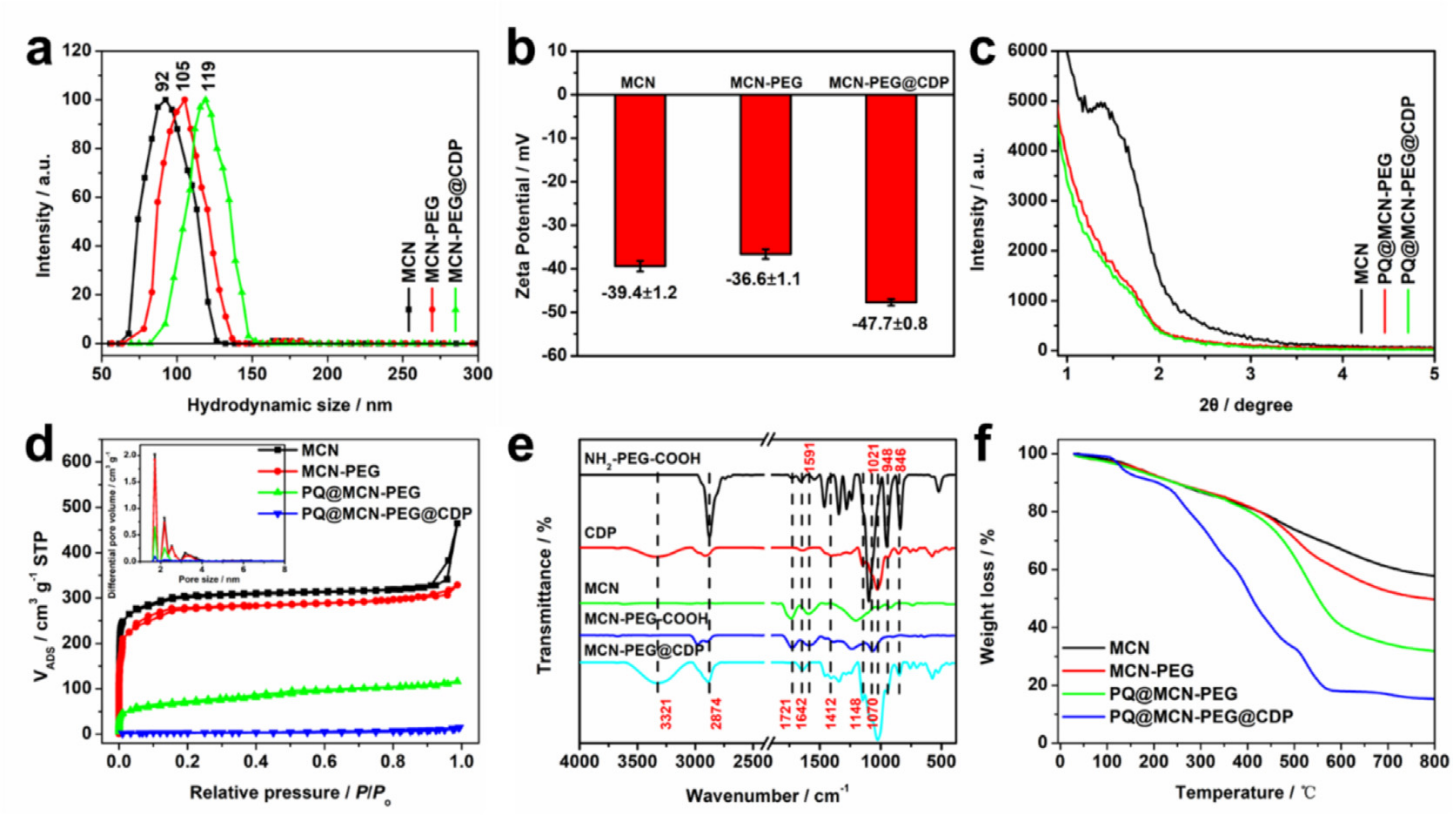
(a) Hydrodynamic sizes, (b) zeta potentials, (c) Small-angle powder XRD patterns, (d) nitrogen adsorption − desorption isotherms (the inset exhibits the pore diameter ranges of counterparts), (e) FTIR spectra, and (f) TGA profiles of PQ@MCN-PEG@CDP at different stages of synthesis.

The mean hydrodynamic sizes of MCN, MCN-PEG, and MCN-PEG@CDP in water were 92, 105, and 119 nm as surveyed by dynamic light scattering (DLS) (Fig. 2a and Table S1), which were consistent with the sizes of the counterpart NPs observed by SEM and TEM photographs, respectively (Fig. 1). The zeta potential of MCN was –39.4 mV resulting from the carboxyl (–COOH) groups on MCN surface, while the zeta potentials of MCN-PEG and MCN-PEG@CDP were –36.6 and –47.7 mV owing to the modification of NH_2_-PEG-COOH (containing negative-charged carboxyl) and coating of CDP (containing negative-charged hydroxyl), respectively (Fig. 2b and Table S1). A wide band at 1591 cm^−1^ in the Fourier transform infrared spectroscopy (FTIR) spectrum of MCN is principally vested in the aromatic C=C stretching vibration on account of the aromatization of MCN (Fig. 2e) [53]. A strong band at 1642 cm^−1^ (amide I band) is vested in the C=O–NH stretching vibration in MCN-PEG based on the amidation reaction between the –COOH of MCN and –NH_2_ of NH_2_-PEG.

For the as-prepared CDP (Fig. S1), the highly water-soluble CDP could be synthesized from α–CD by crosslinking with epichlorhydrin (EP) under basic conditions [48]. The ^1^H NMR, ^13^C NMR, and FTIR spectrum of CDP are appeared in Fig. S2–S4, respectively. A wide band nearby 3321 cm^−1^ is vested in the stretching vibration of O–H, the sharp band at 2874 cm^−1^ is vested in the stretching vibration of antisymmetric CH_2_ (Fig. S4) [49]. The band at 1148 cm^−1^ is assigned to the stretching vibrations of out-of-phase C–C–O in *sec*-alcohols (Fig. S4) [49]. The bands at 1070, 1021, and 948 cm^−1^ are vested in the stretching vibrations of out-of-phase C–C–O in primary alcohols or *sec*-alcohols (Fig. S4) [49]. For MCN-PEG@CDP, two bands at 3321 and 2874 cm^−1^ are vested in the stretching vibration of O–H and antisymmetric CH_2_ in CDP, respectively. The bands at 1070, 1021, and 948 cm^−1^ deriving from the stretching vibrations of out-of-phase C-C-O in CDP are also appeared in MCN-PEG@CDP spectrum (Fig. 2e).

Raman spectrum of MCN appears two scattering peaks at G band 1590 cm^−1^ and D band 1342 cm^−1^ (Fig. S5), indicating the existence of the disordered carbon and the sp^2^-hybridized carbon respectively [50], [51], [52], where the intensity ratio of G/D band is directly proportional to the degree of graphitization. The as-prepared MCN had a large degree of graphitization on the basis of the intensity ratio of G/D band > 1, which means that the graphitic structure existed in MCN not only possesses the hydrophobic characteristic to load drugs [24], [25] but affords the optical absorption performance in the region of NIR [27], [28], [29]. MCN appeared a main small-angle X-ray diffraction (XRD) peak (Fig. 2c), which is the characteristic of mesostructures [23]. The intensity of XRD peak showed a sharp weakness in PQ@MCN-PEG, and almost disappeared in PQ@MCN-PEG@CDP which derived from the padding of MCN pore with PQ loading and coating with CDP gatekeepers (Fig. 2c). The N_2_ adsorption–desorption isotherms of MCN exhibit a type-I model curve with H1 hysteresis loop at relative high pressures and an indiscernible capillary condensation course at relative low pressures (Fig. 2d), which are the characteristics of the micropores, mesopores or interparticle voids [23]. The BET specific surface areas of MCN and MCN-PEG were ca.928 and 876 m^2^ g^−1^, the pore volumes were ca. 0.42 and 0.34 cm^3^ g^−1^ respectively, and the pore diameters were ca.1.7, 2.1, and 2.5 nm via the density functional theory (Fig. 2d and Table 1), which indicated that the decoration of PEG stalks on the MCN surface had almost no influence on the pore sizes of MCN. Upon the loading of PQ and subsequent coating of CDP, both the pore volumes and specific surface areas were sharply decreased owing to the pores of MCN were padded with PQ loading and coated with CDP gatekeepers (Fig. 2d and Table S2). The thermogravimetric analysis (TGA) profiles of MCN, MCN-PEG, PQ@MCN-PEG, and PQ@MCN-PEG@CDP were surveyed in airflow. These NPs at different stages encountered a step-by-step weight loss upon the increase of temperature, while a sharp weight loss in the scope of 450–600 °C and subsequently achieved a plateau (Fig. 2f), which is in line with that of N_2_ adsorption–desorption isotherms.

The loading amount of the technical PQ within MCN-PEG@CDP was determined to be ca. 6.8 % by HPLC according to its standard curve (Fig. S6). The relatively low loading efficiency reflects that PQ loaded in MCN-PEG@CDP was primarily adhered on the pore walls of MCN through the robust π–π effects between MCN (electron enrichment) and PQ (electron deficiency), and few or almost no PQ was retained in the inner cavity of the pores owing to its good water solubility.

### Photothermal performance of MCN-PEG@CDP nanoherbicides

**Fig. 3:**
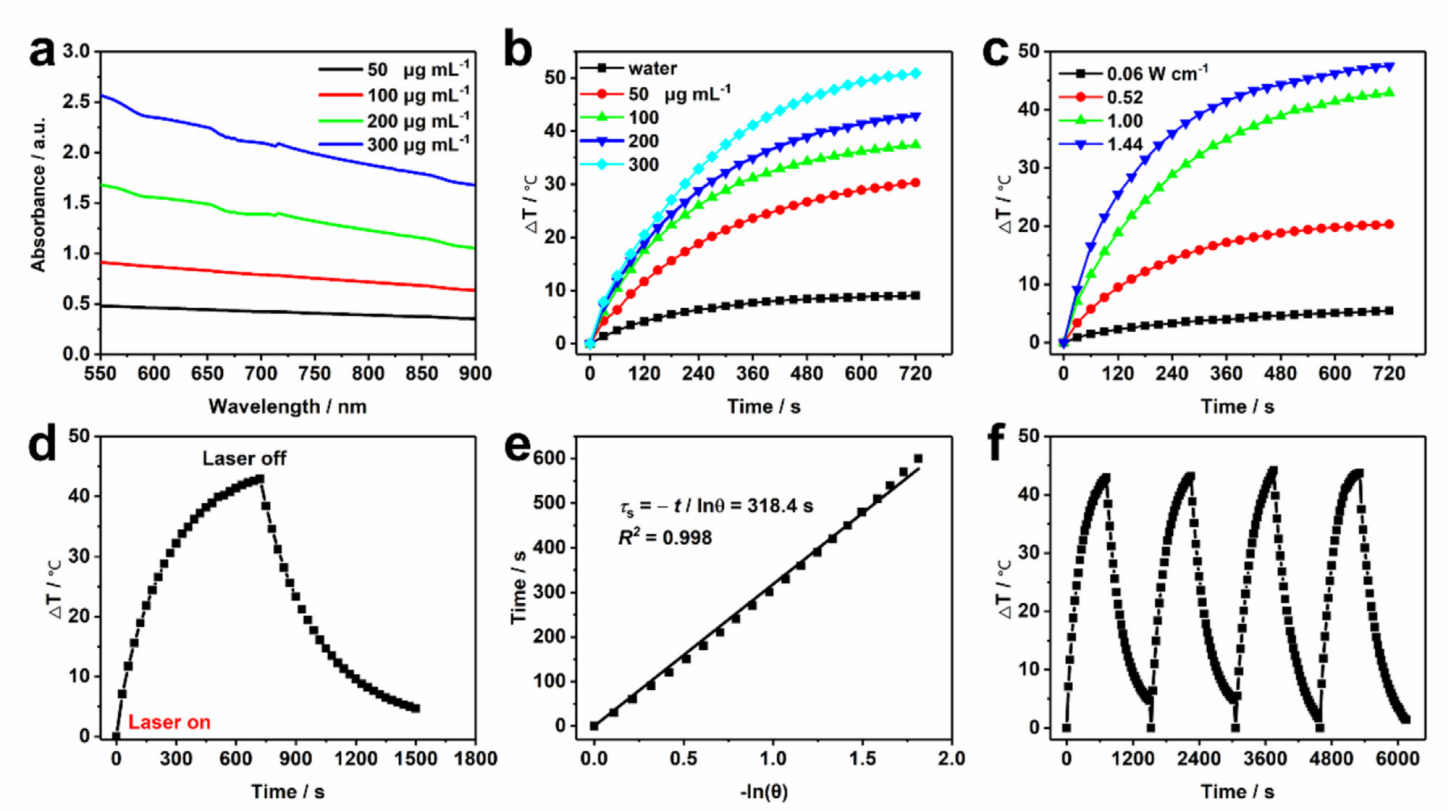
(a) The ultraviolet–visible–NIR of the water dispersions of MCN-PEG@CDP NPs at various contents. Temperature change (ΔT) of the water dispersions of MCN-PEG@CDP NPs (b) at various contents upon the given 808 nm NIR power density (1.00 W cm^−2^), (c) at various power densities upon the given NP content (200 μg mL^−1^). (d) ΔT of the water dispersions of MCN-PEG@CDP (200 μg mL^−1^) at the given 808 nm NIR power density (1.00 W cm^−2^) for 720 s along with a cooling step. (e) Time constant (*τ*_s_) for heat transmission in the system, which was ca. 318.4 s according to the linear time-dependent data (*R*^2^ = 0.998). (f) ΔT of the water dispersions of MCN-PEG@CDP (200 μg mL^−1^) upon ON/OFF cycles at the given 808 nm NIR power density (1.00 W cm^−2^).

MCN-PEG@CDP NPs have obvious broadband absorption, and the near infrared spectrum (NIR) absorbance intensity at 808 nm (except as otherwise noted) increased upon the increase of their concentration (Fig. 3a). The photothermal effect of MCN-PEG@CDP NPs (as a NIR photothermal transducer) were enhanced not only upon the increased NPs concentration at the given NIR power density (Fig. 3b) but upon the elevated NIR power density at the given NPs concentration (Fig. 3c). The photothermal conversion efficiency (PCE) of MCN-PEG@CDP NPs was ca. 40.81 % under 808 nm NIR irradiation (Fig. 3d, e) referring to the calculation formulas in the papers [53], [54], and PCE maintained nearly constant during the 4 cycles of NIR ON/OFF (Fig. 3f). The abundant NIR irradiation energy (52 % of the total solar irradiation energy [46]) could be exploited through the photothermal effect of MCN-PEG@CDP NPs to control the release of trapped PQ.

### Controlled release of the PQ@MCN-PEG@CDP nanoherbicides

**Fig. 4:**
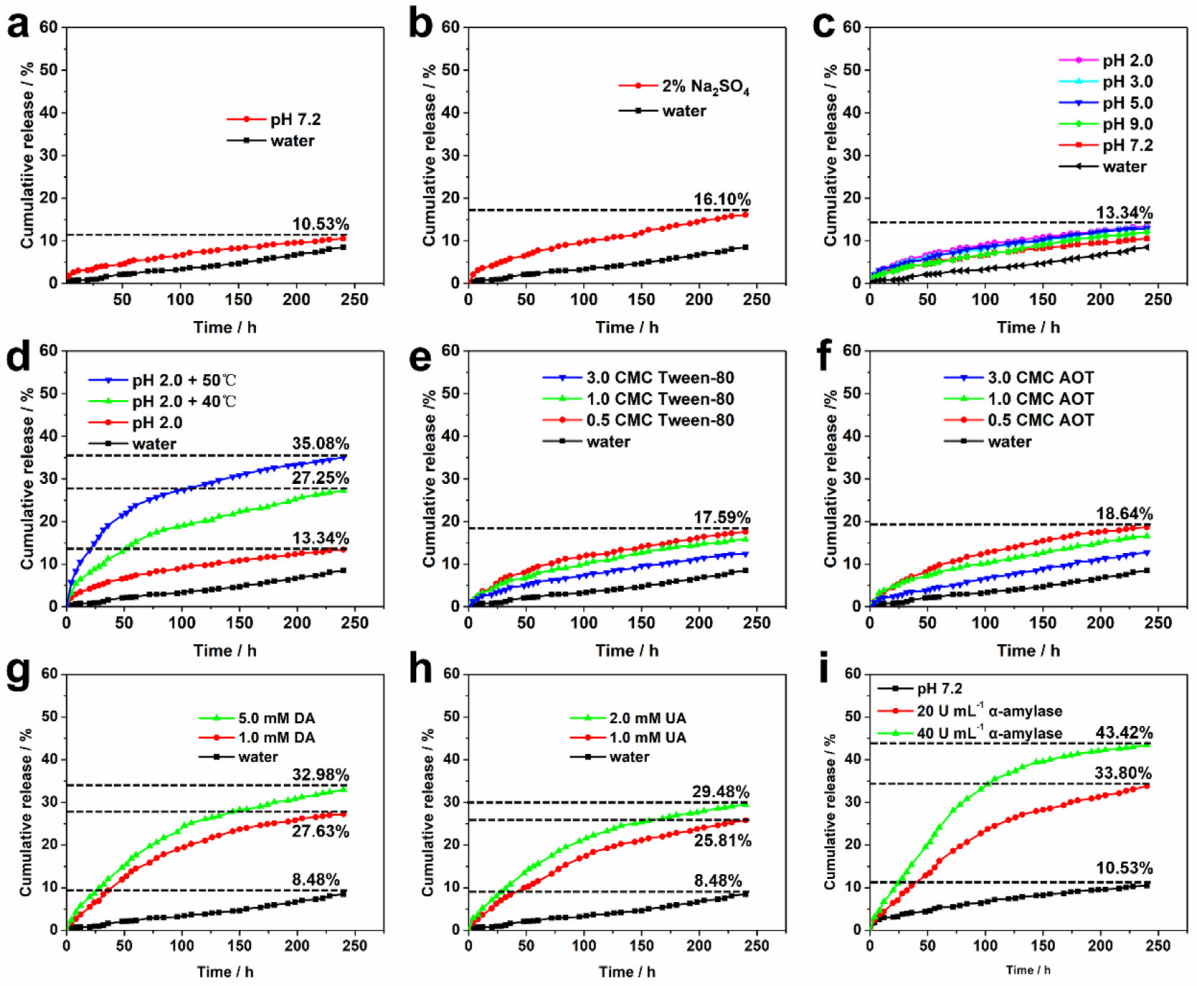
Stimuli-responsive release of the technical PQ from PQ@MCN-PEG@CDP nanoherbicides in water solutions with 0.1 % Tween-80: (a) pH 7.2 PBS buffer system and water; (b) in the existence of 2 % Na_2_SO_4_, with water as control; (c) at various pH; (d) at pH 2.0 + various temperatures; (e, f) Tween-80 (CMC_Tween-80_ = 0.014 g L^–1^) and Aersosol OT (AOT, CMC_AOT_ = 2.5 mM) at various fractions of CMC; (g) decanoic acid (DA); (h) ursolic acid (UA); (i) α–amylase, with pH 7.2 PBS buffer system as control.

The pH environment of various organs in the human body is different. Herein, to guarantee the user safety of the as-prepared nanoherbicides, especially in strongly acidic gastric and alkaline intestinal environments, a broad pH extent of 2.0–9.0 was designed to investigate the technical PQ release from the PQ@MCN-PEG@CDP nanoherbicides. The cumulative release efficiencies (CRE) of the technical PQ from the as-prepared nanoherbicides were very low under the pH extent of 2.0–9.0 (Fig. 4c). After 48 h of pH stimuli, CRE of the technical PQ were only ca. 4.42 % at 9.0, 4.43 % at pH 7.2, and 6.54 % at pH 2.0; Even until day 10, CRE were only ca. 12.00 % at 9.0, 10.53 % at pH 7.2, and 13.34 % at pH 2.0 (Fig. 4c). It is indicated the technical PQ can be barely leakage from PQ@MCN-PEG@CDP at pH 7.2 or pH 9.0 (e.g. the intestinal pH environment of humans), and even at pH 2.0 (e.g. the gastric pH environment of humans). Obviously, CDP nanovalves could not be dethreaded from the MCN surface under a broad pH extent. Even though the PQ@MCN-PEG@CDP nanoherbicides were ingested by humans, these results suggest that the lethal PQ could be barely freed from PQ@MCN-PEG@CDP in the gastrointestinal tract environment. Thus, it is quite different from the usage of adsorbents (e.g. activated charcoal) to inhibit the permeation of technical PQ in the gastrointestinal tract environment in clinical administration against PQ toxicosis [7], [8], [9]. Besides, the emetics and laxatives are commonly served to remove PQ from the gastric area and intestinal canal once PQ is ingested by humans. 2 % Na_2_SO_4_ aqueous solution is one of laxatives commonly used against PQ toxicosis. In the existence of 2 % Na_2_SO_4_ aqueous solution, CRE of the PQ was quite limited, with no obvious increase compared with that in water (Fig. 4b).

In addition, the release of PQ at given high temperature was investigated for mimicking that under NIR irradiation by the photothermal conversion effect. Generally, the air temperatures in nature are not exceed 50 °C, while the release efficiency of PQ is most likely to be enhanced under sunlight because of the remarkable rise of local temperature around MCN-PEG@CDP NPs through the PCE (Fig. 3). The CRE of PQ from PQ@MCN-PEG@CDP were increased with the increased temperature, and reached ca. 27.25 and 35.08 % after 240 h at the temperature of 40 and 50 °C, respectively (Fig. 4d). At high temperature, not only because the host–guest effects (mainly the noncovalent interactions) between the chamber of CD units in CDP and the PEG stalks on the MCN surface were weakened or eliminated to open the CDP gatekeepers, but also the π–π effects between PQ (electron deficiency) and MCN (electron enrichment) were weakened. Both lead to the release of trapped PQ from PQ@MCN-PEG@CDP. It is clear shown the release of the trapped PQ can be realized under sunlight through the PCE of MCN materials.

The hydrophobic inner chamber of α–CD could accommodate different kind of hydrophobic segments or hydrophobic molecules [30], [31]. The ursolic acid (UA), as a pentacyclic triterpenoid, is occurred in a variety of plants [55], [56]. Owing to the hydrophobic framework, UA can be involved in the inner chamber of CD units in CDP, generating the dethreading of CDP from the PEG stalks to release the trapped PQ (Fig. 4h). The CRE of trapped PQ from PQ@MCN-PEG-CDP reached ca. 25.81 and 29.48 % after 240 h at 1.0 and 2.0 mM, respectively. The fatty acids [e.g. decanoic acid (DA)], as an aliphatic compounds, could also cause the dethreading of CDP from the PEG stalks to release the trapped PQ (Fig. 4g). The CRE of trapped PQ from PQ@MCN-PEG-CDP reached ca. 27.63 and 32.98 % after 240 h at 1.0 and 5.0 mM, respectively. Both UA and DA appeared similar binding affinity to CDP. The threading/dethreading of the CDP nanovalves were administrated in a dynamic balance, thus it is clear shown the epicuticular waxes of target plants (containing hydrophobic aliphatic ingredients and cyclic ingredients [40], [41]) can be as competitors to regulate and control the release of trapped PQ from PQ@MCN-PEG@CDP.

For the same reason, Tween-80 and AOT, similar to UA and DA, could be also involved into the inner chamber of CD units in CDP [32]. However, in the existence of two surfactants, no obvious release of trapped PQ was observed (Fig. 4e, f). Below CMC, the CRE of the trapped PQ appeared a slight enhance with the growth of surfactants content; while above CMC, a gentle decrease with the growth of surfactants content resulting from the surfactant aggregates formation. The results shown that PQ@MCN-PEG@CDP nanoherbicides appeared good stability without obvious early leakage when Tween-80 or AOT additives are present.

α–Amylase could be produced from some of fungi and plants, which could hydrolyze CD units into glucopyranose subunits [42], [43], [44], [45], [57]. In the existence of α–amylase, CDP gatekeepers could be hydrolyzed and opened to release the trapped PQ from PQ@MCN-PEG@CDP (Fig. 4i). The CRE of the technical PQ increased with the growth of α–amylase content, and reached ca. 33.80 and 43.42 % after 240 h at 20 and 40 U mL^−1^, respectively.

It is clear that the PQ@MCN-PEG@CDP nanoherbicides could regulate the release of trapped PQ to enhance the efficacy and bioavailability toward target plants through multi-stimuli responses (e.g. sunlight, competitors, and enzymes). While, the overall CRE of trapped PQ from the PQ@MCN-PEG@CDP nanoherbicides were low and did not exceed 50 %. It is possibly because that a large amount of the trapped PQ was forcefully adhered on the surface of MCN pore walls via the robust π–π effects between the technical PQ (electron deficience) and MCN (electron enrichment) rather than in the inner cavity of the MCN pores. Fascinatingly, the robust π–π effects between the trapped PQ and MCN pore walls, and the host–guest effects between the chamber of α–CD units in CDP and PEG stalks on the MCN surface were almost no disturbed under pH 2.0–9.0 region, purgative agent 2 % Na_2_SO_4_, Tween-80 and AOT additives, which could be developed for a promising bio-friendly PQ formulation.

### Spreading and retention of MCN-PEG@CDP NPs on hydrophobic surface

Most of plant leaves are hydrophobic with different kinds of micro/nanostructured processus mastoideus consisting of epicuticular wax congeries which contain cyclic compounds (flavonoids, triterpenoids, etc) and aliphatic compounds (fatty acids, wax esters, etc) [40], [41]. After spraying, the fluid drop of pesticides should deposition on the target leaf surfaces, and the bioactive components could be released and delivered to the sites of biological target, and finally kill the pests, pathogens or weeds [13]. While, more than 50 % of pesticides are wasted upon spraying, not only because of undesirable splashing or bouncing behavior of water drops on water-resistant surface of plant leaves [58], [59], [60], [61], but because of the inescapable natural factors (gravity, rainwater, wind, etc). Once the technical PQ contacts with the soil or other organic substances, it will lose biological activity and ineffective for the target plants. Although organic solvents are not employed for the application of PQ formulations in the field, it is necessary to add surfactants to boost the final spreading and retention of water drops of nanopesticides on the hydrophobic leaf surfaces [59], [15], [60], [61]. Considering that freshly harvested plant leaves are prone to dehydration and deformation upon contact angle measurement, glass slides decorated by octadecyltrichlorosilane (OTS) were selected as model hydrophobic leaves. The contact angles of water drops with the MCN, MCN-PEG, and MCN-PEG@CDP in the present of Tween-80 or AOT were surveyed on the model hydrophobic leaf surface.

**Fig. 5:**
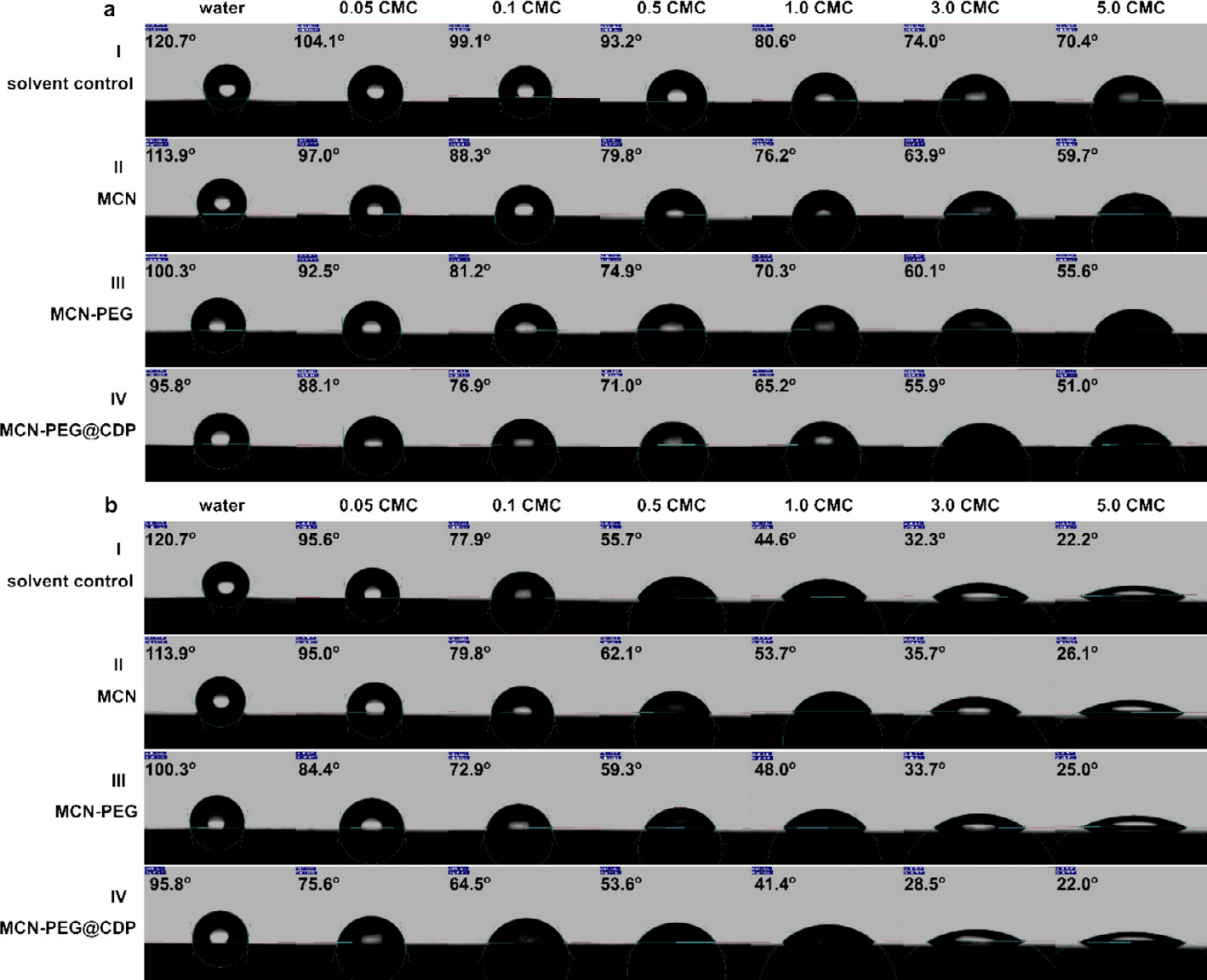
Contact angles of the water drops with MCN, MCN-PEG, and MCN-PEG@CDP NPs on the hydrophobic slide surfaces (*n* = 3) in the existence of (a) Tween-80 and (b) AOT at various fractions of CMC, respectively. The counterparts without NPs as solvent control.

In the present of the frequently-used additive Tween-80 with diverse fractions of critical micelle concentration (CMC) (*X*_CMC-T_; CMC_Tween-80_ = 14 mg L^–1^), the water drops appeared a reduction in contact angle on the hydrophobic slide surface with the growth of *X*_CMC-T_ from 120.7° in pure water (*X*_CMC-T_ = 0) to 70.4° at *X*_CMC-T_ = 5.0 (Fig. 5a, Fig. S8, and Table S4). The water drops with MCN appeared a similar alteration in contact angle to those only with Tween-80. While the water drops with MCN-PEG appeared a further reduction, which was reduced from 100.3° at *X*_CMC-T_ = 0 to 55.6° at *X*_CMC-T_ = 5.0 (Fig. 5a, Fig. S8, and Table S4). It is shown the modification of PEG polymer on MCN surface was beneficial to the wettability and spreading of MCN-PEG on the hydrophobic surface. Furthermore, in the case of MCN-PEG@CDP, the contact angle markedly reduction compared with that uncapped MCN-PEG, which was decreased to 88.1° at *X*_CMC–T_ = 0.05 and 51.0° at *X*_CMC–T_ = 5.0 on the hydrophobic slide surface (Fig. 5a, Fig. S8, and Table S4). A relatively conspicuous difference manifests that the abundant hydroxyl (−OH) of the CDP supramolecular were beneficial to the wettability and spreading of the MCN-PEG@CDP NPs on the hydrophobic surfaces in the existence of Tween-80. It is possible the MCN-PEG@CDP NPs can shape hydrogen bonds and hydrophobic effects with epicuticular wax compounds existed on the plant leaf surface.

AOT (CMC_AOT_ = 2.5 mM), due to its special structural properties, possesses the speedy diffusion movement to the liquid–solid and/or air–liquid interface, and therefore has the powerful capacity to weaken the interfacial tension on the surface of fresh generated interfaces [62]. In the present of AOT with diverse fractions of CMC (*X*_CMC-A_; CMC_AOT_ = 2.5 mM), the counterparts suffered a remarkable reduction in the contact angle on the hydrophobic slide surface with the elevation of *X*_CMC-A_. The contact angle of the water drops only with AOT reduced from 120.7° at *X*_CMC-A_ = 0 to 22.2° at *X*_CMC-A_ = 5.0 (Fig. 5b, Fig. S8, and Table S5). The water drops only with AOT can be well infiltrated on the hydrophobic interface at *X*_CMC-A_ = 0.9 [62]. With addition of AOT, the water drops with MCN, MCN-PEG, and MCN-PEG@CDP NPs appeared a similar alteration of contact angle on the hydrophobic slide surface compared with that only with AOT, but the water drops with MCN-PEG@CDP in contact angle appeared better wettability and spreading than those with MCN and MCN-PEG, which was 75.6° at *X*_CMC-A_ = 0.05 and 22.0° at *X*_CMC-A_ = 5.0 (Fig. 5b, Fig. S8, and Table S5). The magnitude of the reduction in contact angle for the water drops of MCN-PEG@CDP with AOT was much larger than that with Tween-80, which might be that NPs with nanoscale highly dispersed hydrophilic interfaces (the zeta potential of − 47.7 mV) can conveniently generate dynamic “composite” hydrophilic nanostructured papillae on the hydrophobic slide surface (120.7° in pure water) in the existence of AOT. It is clearly indicated that the existence of AOT and the coating of CDP gatekeepers could dramatically ameliorate the wettability and spreading of NPs on the hydrophobic surfaces, which is consistent with our previous report [47].

**Fig. 6:**
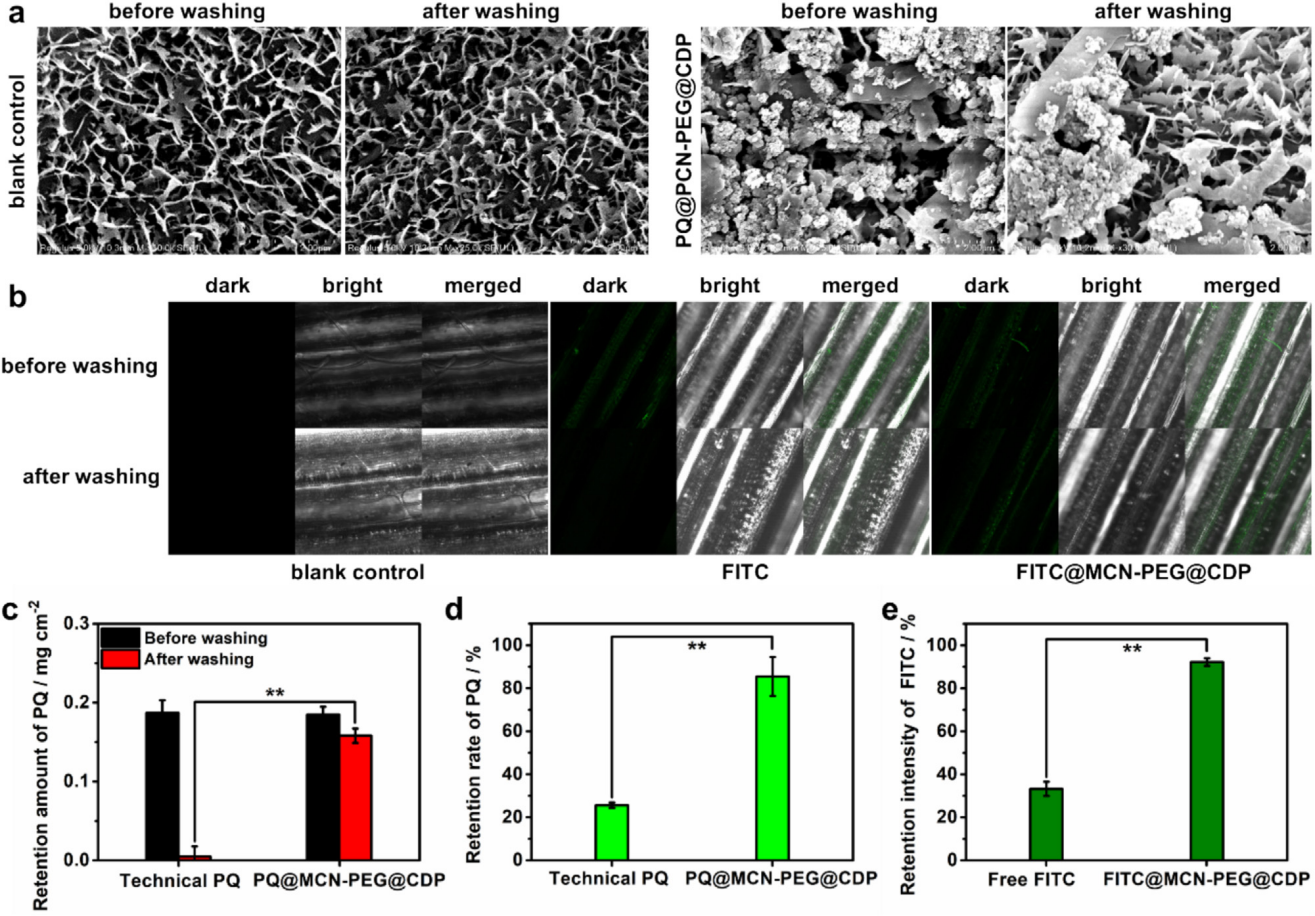
(a) SEM photographs of PQ@MCN-PEG@CDP NPs, (b) Laser confocal imaging of FITC@MCN-PEG@CDP NPs, (c, d) The retention amount and retention rate of technical PQ and PQ@MCN-PEG@CDP NPs (converted into the PQ amount), (e) Retention intensity of free FITC and FITC@MCN-PEG@CDP (converted into the FITC intensity) on outdoor weed (*Cynodon dactylon*) leaf surface before and after washing with water, respectively. Each experiment was performed three times (*n* = 3). The data are the mean values ± standard errors. Comparisons of the leaf retention amount and fluorescence image intensity results were analyzed using one-way analysis of variance (ANOVA) and Duncan’s multiple range test. One star in graphs means that the comparison is significant at *P* < 0.05, and two stars means that the comparison is significant at *P* < 0.01.

The performance of retention and washing resistance of PQ formulations on weed leaf surfaces after spraying is an important factor to affect the utilization rate of the technical PQ. Firstly, the adhesion ability of PQ@MCN-PEG@CDP NPs on outdoor weed (*Cynodon dactylon*) leaf surfaces was observed by SEM before and after washing with water at a 60° bevel. It could be seen the weed leaf surfaces were covered with many irregular folds, and porrect wax island structures consisted by the foliage surface cuticle, epidermal cells and stomatal guard cells (Fig. 6a, left), which caused the lotus foliage effect. After mimicking the rain washing, it shown that there was still a large amount of PQ@MCN-PEG@CDP NPs retained on the weed leaf surface (Fig. 6a, right). The retention rate of PQ@MCN-PEG@CDP (converted into PQ amount) after washing was 3.33 times higher than that of technical PQ, which shown a significant difference (^**^P < 0.01) (Fig. 6c, d and Table S6). Furthermore, fluorescence imaging was selected to verify the retention and washing resistance of MCN-PEG@CDP nanocarriers using FITC labeling (denoted as FITC@MCN-PEG@CDP). It shown that FITC@MCN-PEG@CDP NPs with green fluorescence could still be sighted abundantly on the weed surfaces after rain washing with water compared with free FITC (Fig. 6b). The retention intensity of FITC@MCN-PEG@CDP (converted into FITC intensity) after rain washing was 2.77 times higher than that of free FITC, which shown a significant difference (^**^P < 0.01) (Fig. 6e, Table S7). The results indicate the PQ@MCN-PEG@CDP nanoherbicides have good deposition and retention on outdoor weed leaf surfaces. The grounds for the enhanced retention and washing resistance of PQ@MCN-PEG@CDP NPs may be that I) some NPs could be embedded in the cracks or creases of the waxy layer structure (Fig. 6a); II) the host–guest effects between the chambers of CD in CDP that did not thread with PEG stalks and epicuticular wax compounds (fatty acids, triterpenoids, etc) existed on the leaf surface (Fig. 4g, h); III) the hydrogen bond interaction, or the topological structures were formed between the waxy layer and PQ@MCN-PEG@CDP NPs [63].

### Safety evaluation of PQ@MCN-PEG@CDP nanoherbicides *in vitro* cytotoxicity and *in vivo* mouse model

**Fig. 7:**
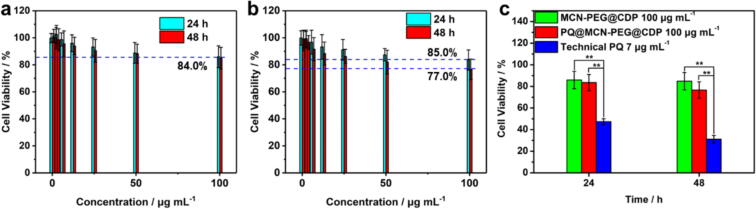
Cell viability of (a) MCN-PEG@CDP, (b) PQ@MCN-PEG@CDP, and (c) MCN-PEG@CDP and PQ@MCN-PEG@CDP at 100 μg  mL^−1^ (same as 7 μg  mL^−1^ of the technical PQ) compared with technical PQ at 7 μg  mL^−1^ for cultivation 24 and 48 h with human LO-2 cells. The data are the mean values ± standard errors. Comparisons of the cell viability were analyzed using one-way analysis of variance (ANOVA) and Duncan’s multiple range test (*n* = 6, *P < 0.05, ^**^P < 0.01).

The toxicity of PQ@MCN-PEG@CDP nanoherbicides on human normal hepatic cells (LO-2) were firstly evaluated considering the damage on the liver after PQ intake. The MCN-PEG@CDP (100 μg mL^−1^) nanocarriers without PQ loading appeared cell viability of ca. 86.0 and 85.0 % upon LO-2 cells after 24 and 48 h, respectively (Fig. 7a, b), which shown that the nanocarriers have low toxicity. Satisfactorily, the PQ@MCN-PEG@CDP (100 μg mL^−1^, same as of 7 μg mL^−1^ of the technical PQ) nanoherbicides also appeared cell viability of ca. 84.0 and 77.0 % toward LO-2 cells after 24 and 48 h respectively (Fig. 7a, b). The low cytotoxicity of as-prepared nanoherbicides was similarly to that of nanocarriers without PQ loading. While the technical PQ at the usage of 7 μg mL^−1^ appeared cell viability of ca. 47.4 and 31.2 % toward LO-2 cells after 24 and 48 h, respectively (Fig. 7c and S7). The cell viability of PQ@MCN-PEG@CDP (same as 7 μg mL^−1^ of the technical PQ) were 1.81 and 2.72 times higher than that of technical PQ after 24 and 48 h respectively, which shown a significant difference (^**^P < 0.01) (Fig. 7c, Table S8). The result manifested that the as-prepared PQ formulations appeared lower damage to LO-2 cells compared with technical PQ.

**Fig. 8:**
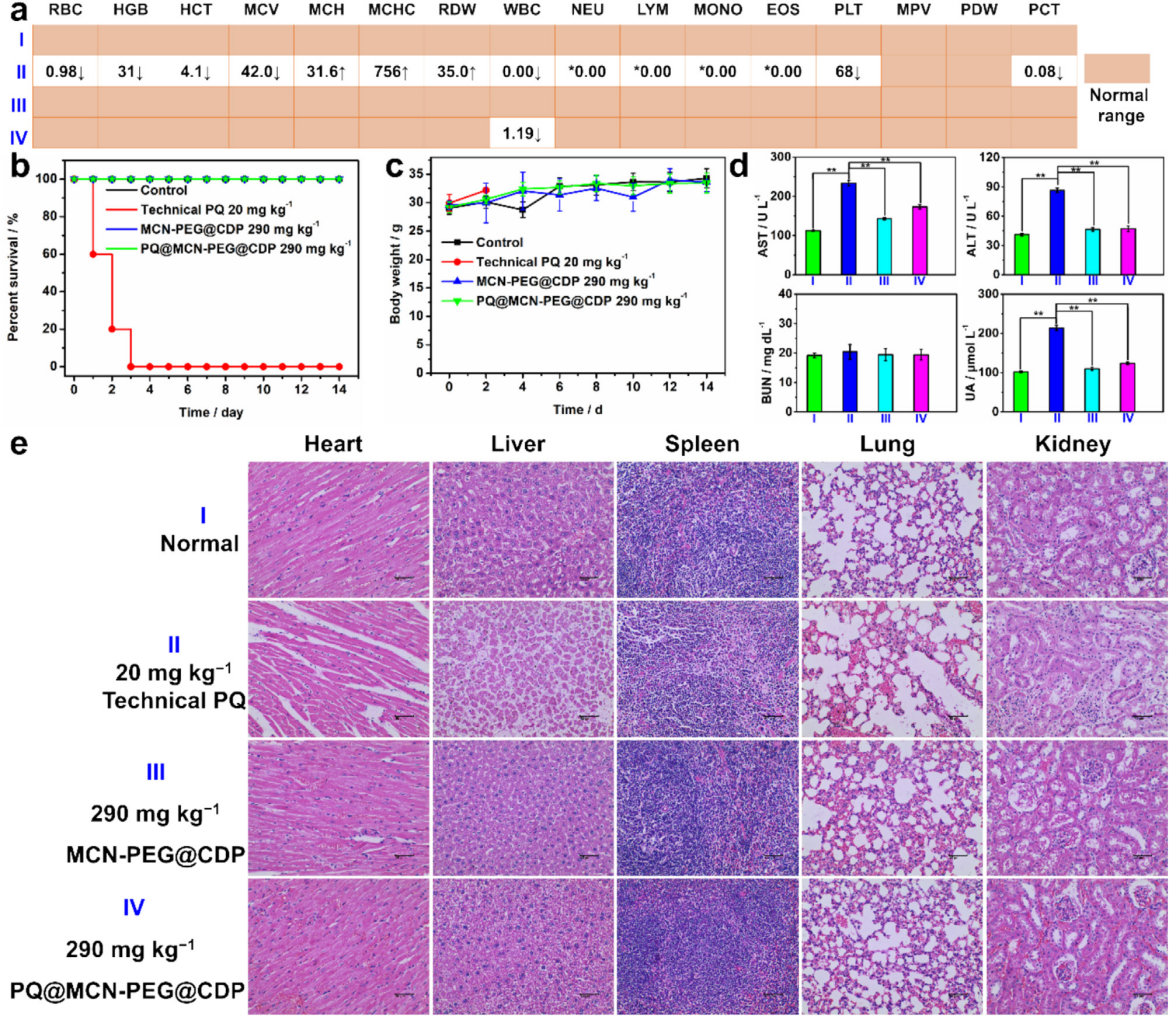
(a) The hematology analysis (*n* = 3), (b) Survival rate (*n* = 6), (c) change of body weights of 6-week-old female C57BL/6 mice (*n* = 6), (d) Quantitative analysis of liver and renal function biomarkers (ALT, AST, BUN, and UA) in the blood of the mice (*n* = 3, *P < 0.05, ^**^P < 0.01), (e) H&E staining images of the major organs (lung, liver, spleen, kidney and heart) of the mice (*n* = 3) after intragastrical administration with PQ (20 mg kg^−1^), MCN-PEG@CDP (290 mg kg^−1^) and PQ@MCN-PEG@CDP (290 mg kg^−1^, same as the technical PQ dose of 20 mg kg^−1^) after 14 days unless the technical PQ group died before that date. All of scale bars are 50 μm. The data are the mean values ± standard errors. Comparisons of the liver and renal function biomarkers were analyzed using one-way analysis of variance (ANOVA) and Duncan’s multiple range test (*n* = 3, *P < 0.05, ^**^P < 0.01).

Furthermore, A mouse model *in vivo* was employed to test the safety of the as-prepared PQ formulations. Female C57BL/6 mice (6 weeks old) were intragastrically administrated with technical PQ at the dose of 20 mg kg^−1^, MCN-PEG@CDP at 290 mg kg^−1^, and PQ@MCN-PEG@CDP at 290 mg kg^−1^ (same as the PQ dose of 20 mg kg^−1^) on the first day, and their body weights and survival rates were subsequently recorded for escalation evaluation. The blood was collected for hematology analysis and biochemical assay. The main parameters of the treated groups with MCN-PEG@CDP and PQ@MCN-PEG@CDP for hematology analysis were in the normal range (similar to that in the control group), while that of technical PQ-treated group appeared significant anomaly (Fig. 8a). The levels of alanine transaminase (ALT) and aspartate aminotransferase (AST) as the hepatic damage biomarkers and uric acid (UA) as the renal function biomarker in the treated groups with MCN-PEG@CDP and PQ@MCN-PEG@CDP appeared significant difference compared with that of technical PQ-treated group (^**^P < 0.01), suggesting a long-term liver and kidney damage by the technical PQ (Fig. 8d) [64], [65]. While, either the technical PQ or PQ@MCN-PEG@CDP did not affect the levels of blood urea nitrogen (BUN) in the serum of the mice (Fig. 8d), suggesting that the renal function might be preserved in part [64], [65]. A 100 % mortality rate of the mice generated after 3 days in the treated groups with technical PQ (20 mg kg^−1^) (Fig. 8b); All of the mice survived after treatment of MCN-PEG@CDP (290 mg kg^−1^) and PQ@MCN-PEG@CDP (290 mg kg^−1^, same as the PQ dose of 20 mg kg^−1^) during the period of 14 days (Fig. 8b). The body weights of mice after treatment of MCN-PEG@CDP and PQ@MCN-PEG@CDP showed no difference over the period of 14 days (Fig. 8c). The histological analysis of the main organs (heart, liver, spleen, lung, and kidney) in mice for each group were operated with H&E staining (Fig. 8e). In the treated groups with technical PQ, obvious hemorrhage and inflammation were observed in the liver, spleen, lungs, heart, and kidneys. Moreover, atrophy of the hepatocytes around the central vein of the liver, widespread thickening of alveolar septum in lungs, obviously swelled myocardial cells in heart, as well as vacuolar degeneration in kidney were also observed. Attractively, the pathological changes and morphology of the organs in the treated groups with MCN-PEG@CDP and PQ@MCN-PEG@CDP appeared no obviously difference compared with the control group (Fig. 8e).

The results shown that the PQ@MCN-PEG@CDP nanoherbicides revealed a good safety on mice via oral administration, as proved at the levels of cells, hematology indicators, biochemical biomarkers, tissue histology, survival life, and physical growth, which signified that the as-prepared PQ formulations were bio-friendly for users.

### Herbicidal efficacy of PQ@MCN-PEG@CDP nanoherbicides

**Fig. 9:**
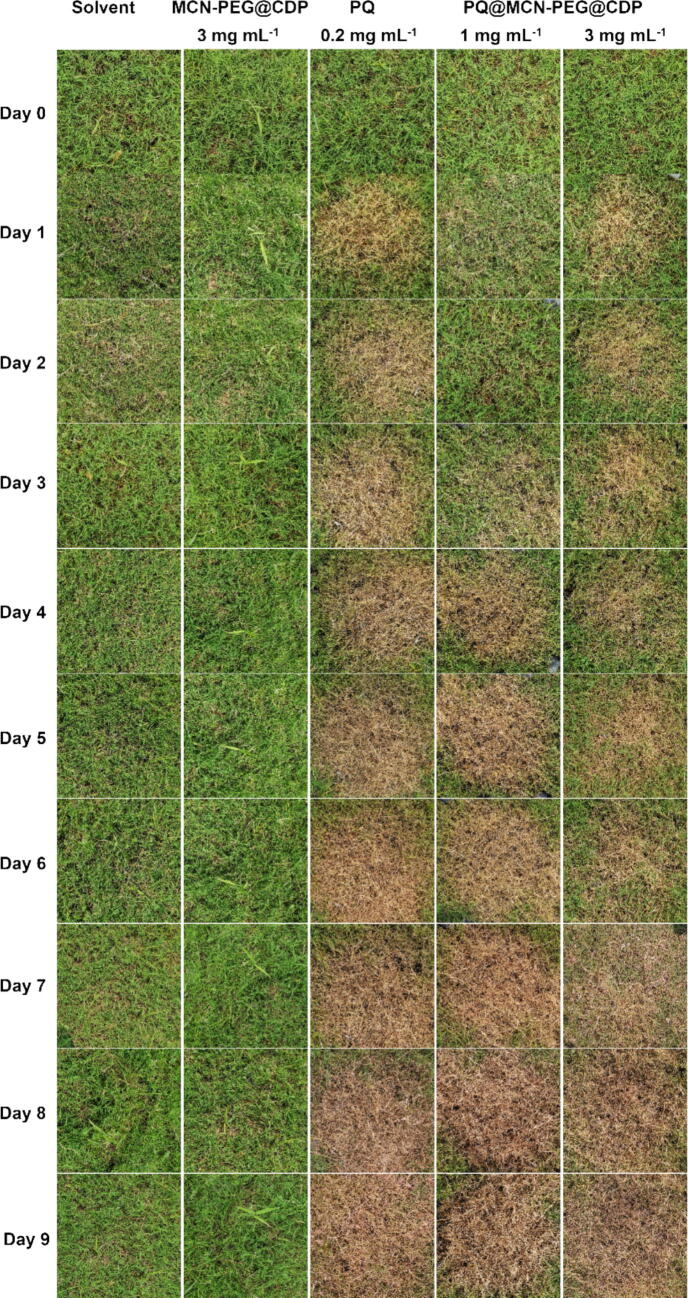
Control efficacy of the PQ@MCN-PEG@CDP nanoherbicides with 1.0 and 3.0 mg mL^−1^ (same as 0.07 and 0.2 mg mL^−1^ of the technical PQ, respectively) after spraying upon outdoor weeds (*Cynodon dactylon*) under sunlight over time (*n* = 3), compared with solvent with 1 % Tween-80, MCN-PEG@CDP, and technical PQ (0.2 mg mL^−1^).

Under the condition of sunlight and O_2_, the technical PQ has high and non-selective herbicidal activity, which will cause rapid decolorization, withering, and death of plant green tissues owing to the disruption of photosynthesis and chlorophyll synthesis. To measure the weed control of the PQ@MCN-PEG@CDP nanoherbicides against *Cynodon dactylon* outdoors, the control efficacies of the technical PQ, MCN-PEG@CDP, and PQ@MCN-PEG@CDP were surveyed out of doors with water as control (Fig. 9 and Table S9). The control efficacies could not be observed in control and MCN-PEG@CDP after spraying. In the treated groups with the technical PQ (0.2 mg mL^−1^), the control efficacy against weeds enhanced gradually over time. The herbicidal efficacies acquired ca. 64.67 % after 2 days, ca. 82.94 % after 4 days, and ca. 98.55 % after 9 days (Fig. S9 and Table S9). In the treated groups with the PQ@MCN-PEG@CDP nanoherbicides, the weeds became decoloration with 1.0 mg mL^−1^ at the third day of spraying, and with 3.0 mg mL^−1^ at the first day of spraying. The control efficacies were both amplified with the growing usage of the nanoherbicides and persistent release of trapped PQ from the nanoherbicides (Fig. 9 and Table S9). The control efficacy was ca. 26.18 % with 1.0 mg mL^−1^ (same as 0.07 mg mL^−1^ of the technical PQ) at the third day of spraying, and achieved ca. 59.61 and 86.46 % after 4 and 6 days of spraying, respectively (Fig. S9 and Table S9). While the control efficacy was ca. 25.08 % with 3.0 mg mL^−1^ (same as 0.2 mg mL^−1^ of the technical PQ) at the first day of spraying, and achieved ca. 79.66 and 86.93 % after 4 and 6 days of spraying, respectively (Fig. S9 and Table S9).

At the third day of spraying, the as-prepared nanohebicides with 3.0 mg mL^−1^ (same as 0.2 mg mL^−1^ of the technical PQ) appeared comparable control efficacy to the technical PQ (0.2 mg mL^−1^); while with 1.0 mg mL^−1^ (same as 0.07 mg mL^−1^ of the technical PQ) appeared comparable control efficacy to technical PQ (0.2 mg mL^−1^) after 6 days of spraying (Fig. S9 and Table S9). It is evident the PQ@MCN-PEG@CDP nanoherbicides with low content of PQ loading could still effectively maintain herbicidal activity and prevent the harm of the high PQ levels to humans, insects, and environment. Compared with traditional PQ formulations, the PQ@MCN-PEG@CDP nanoherbicides may practicably improve biosafety from a long-term point of view to meet the low-cost and bio-friendly challenge in agricuture based on the efficient deposition and delivery performance towards target plants.

### Stability of PQ@MCN-PEG@CDP nanoherbicides

**Fig. 10:**
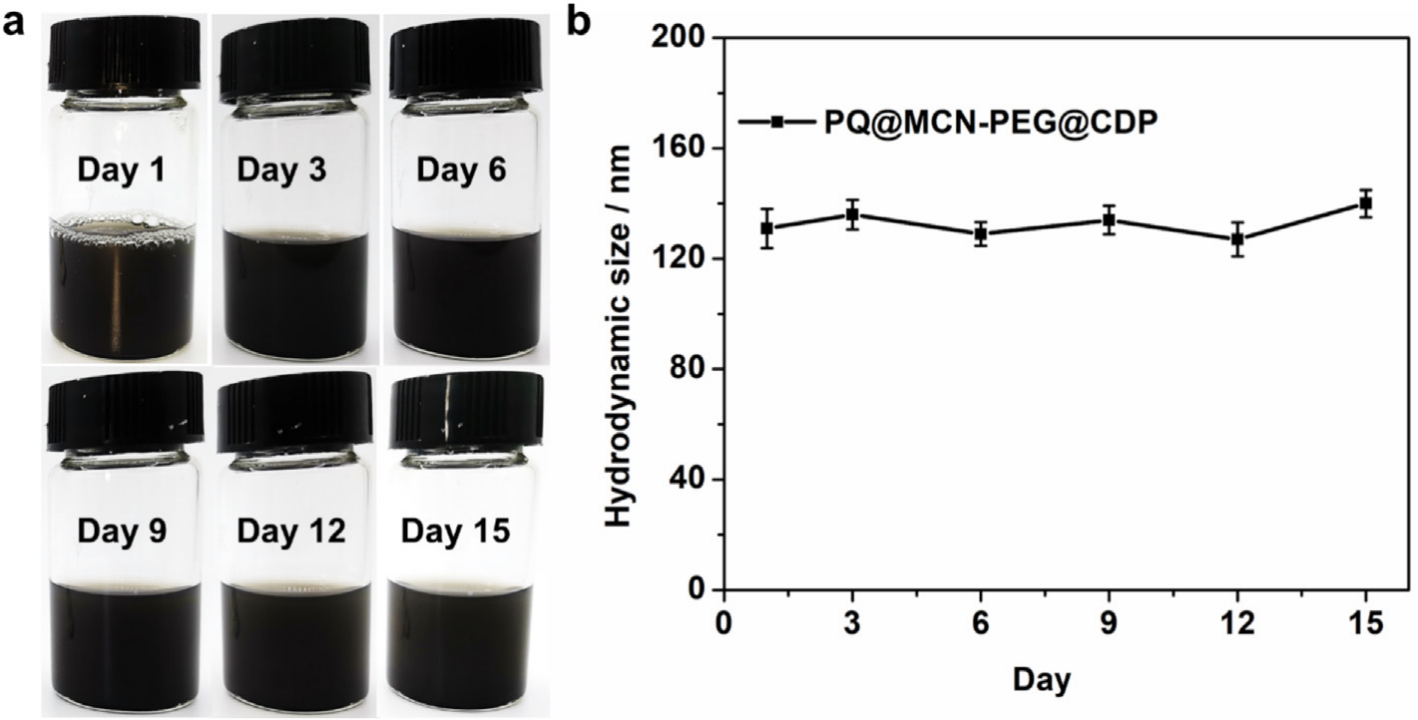
(a) the images and (b) hydrodynamic sizes of PQ@MCN-PEG-CDP nanohebicides in water solution with 1 % Tween-80 for various time period (*n* = 3). The data are the mean values ± standard errors.

Storage stability and shelf life are important indicators for the application of as-prepared PQ@MCN-PEG@CDP nanoherbicides. Obviously, the PQ formulations could enduringly remain dispersibility without aggregation for at least 15 days (Fig. 10a) and appeared almost no premature release of trapped PQ for at least 11 days in aqueous solution with the existence of additives [Tween-80 (Fig. 4g) or AOT (Fig. 4h)]. Their hydrodynamic sizes could maintain unvaried as proved by DLS (Fig. 10b and Table S3). The results indicated that PQ@MCN-PEG@CDP nanoherbicides have good storage stability and shelf life during the actual application.

### Biosafety of PQ@MCN-PEG@CDP nanoherbicides on non-target species

**Fig. 11:**
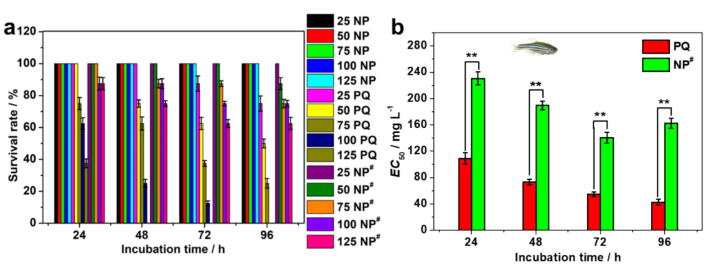
(a) Survival rates and (b) *EC*_50_ value of MCN-PEG@CDP [denoted as NP], technical PQ and PQ@MCN-PEG@CDP [denoted as NP**^#^**] at different PQ dose (same as 25, 50, 75, 100, and 125 mg L^–1^ of the technical PQ) against zebrafish over time. The data are the mean values ± standard errors. Comparisons of the *EC*_50_ value were analyzed using one-way analysis of variance (ANOVA) and Duncan’s multiple range test (*n* = 8 × 3, *P < 0.05, ^**^P < 0.01).

It is necessary to evaluate the biosafety of the PQ@MCN-PEG@CDP nanoherbicides in practical application. The excessive pesticides inevitably contact with water or intake by beneficial insects. Firstly, we selected zebrafish (*D. rerio*, a model species) in water for biosafety evaluation. The technical PQ, MCN-PEG@CDP [denoted as NP], and PQ@MCN-PEG@CDP nanoherbicides [denoted as NP**^#^**] were dispersed in water solution with 1 % Tween-80. After 96 h of incubation, the survival rates of MCN-PEG@CDP NPs remained 100 %, which indicated that the MCN-PEG@CDP without PQ loading nearly have no toxic against zebrafish (Fig. 11, S10 and Table S10-S11). Technical PQ and PQ@MCN-PEG@CDP showed time- and dosage-dependent toxicity against zebrafish (Fig. 11, S10 and Table S10). However, PQ@MCN-PEG@CDP exhibited much higher biosafety than technical PQ under the same conditions. The survival rates of PQ@MCN-PEG@CDP against zebrafish were relatively high after 24 h of incubation and reached ca. 87.50 ± 3.79 % at the PQ dosage of 125 mg L^–1^, while that of the technical PQ were ca. 37.50 ± 2.73 % (Fig. 11a and Table S10). The survival rates of PQ@MCN-PEG@CDP reached ca. 75.00 ± 1.85 % at the PQ dosage of 125 mg L^–1^ after 48 h of incubation (even after 96 h of incubation, reached ca. 62.50 ± 3.88 %), while that of the technical PQ were ca. 0 under the same conditions (Fig. 11a and Table S10). The *EC*_50_ values of PQ@MCN-PEG@CDP against zebrafish were determined to be 189.579 and 162.421 mg L^–1^ after 48 and 96 h of incubation, while that of technical PQ were 73.154 and 42.584 mg L^–1^ at the corresponding incubation time, which are 2.59 and 2.74 times higher than that of technical PQ, respectively (Fig. 11b and Table S11). These results confirmed that the biosafety of PQ@MCN-PEG@CDP against zebrafish were much higher than that of technical PQ and showed very significant differences (^**^P < 0.01).

**Fig. 12:**
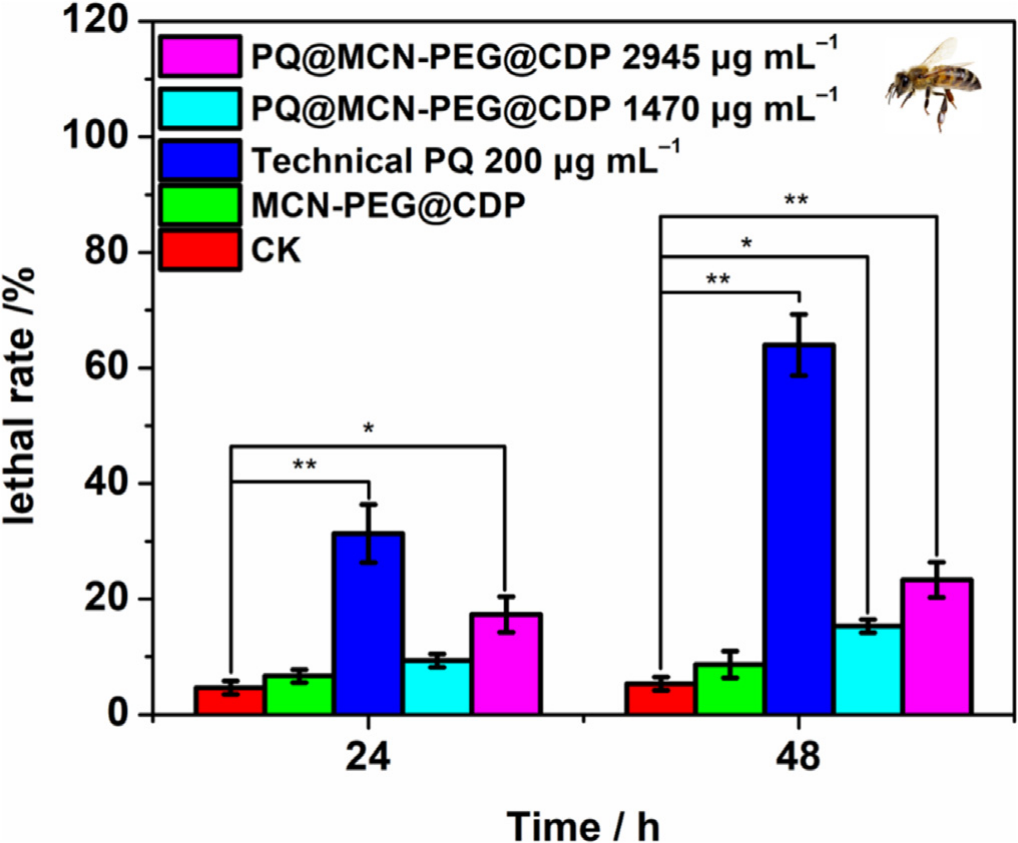
Lethal rate of PQ@MCN-PEG@CDP nanoherbicides to honeybees (*Apis mellifera L.*) at 1470 and 2945 μg mL^−1^ (same as 100 and 200 μg mL^−1^ of the technical PQ, respectively) compared with solvent control (CK), MCN-PEG@CDP (2940 μg mL^−1^), and the technical PQ (200 μg mL^−1^) over time. The data are the mean values ± standard errors. Comparisons of the lethal rates were analyzed using one-way analysis of variance (ANOVA) and Duncan’s multiple range test (*n* = 50 × 3, *P < 0.05, ^**^P < 0.01).

Secondly, we selected honeybees (*Apis mellifera L.,* a beneficial pollinator) as non-target insect for biosafety evaluation. The lethal percentages of honeybees were ca. 17.33 and 23.33 % at 24 and 48 h respectively for the group of PQ@MCN-PEG@CDP with 2945 μg mL^−1^ (same as 200 μg mL^−1^ of the technical PQ) (Fig. 12, Table S12); In the group of technical PQ with 200 μg mL^−1^, lethal percentages were attained ca. 31.33 and 64.00 % at 24 and 48 h (Fig. 12, Table S12), which are 1.81 and 2.74 times higher than that of PQ@MCN-PEG@CDP, respectively (Fig. 12). Compared with blank control (CK), the technical PQ with 200 μg mL^−1^ appeared extremely significant difference (^**^P < 0.01) at 24 and 48 h; the as-prepared PQ@MCN-PEG@CDP nanoherbicides with 1470 μg mL^−1^ (same as 100 μg mL^−1^ of the technical PQ) appeared no significant difference (*P* > 0.05) at 24 h, and significant difference (*P < 0.05) at 48 h (Fig. 12); while with 2945 μg mL^−1^ (same as 200 μg mL^−1^ of the technical PQ) appeared significant difference (*P < 0.05) at 24 h, and extremely significant difference (^**^P < 0.01) at 48 h (Fig. 12). The results manifested PQ@MCN-PEG@CDP nanoherbicides have lower harm on honeybees compared with the technical PQ at the given contents. However, more related toxicity pointer and even long-term toxicity tests are still required to assess the biosafety of PQ@MCN-PEG@CDP nanoherbicides to non-target species before it can be used as a bio-friendly PQ formulation in agriculture.

## Conclusions

In summary, one intelligent and bio-friendly CDP-gated mesoporous carbon nanoparticle (MCN) nanoherbicides for enhanced paraquat delivery were constructed. The capping of CDP gatekeepers was fastened via host–guest effects between the chamber of α–CD units in CDP and PEG stalks on the MCN surface, which can not only prevent PQ early leakage, but facilitate the spreading and retention of NPs on hydrophobic leaf surface based on the improvement of wettability and adhesion. MCN as the nanocarriers could also be served as NIR photothermal transducers. The as-prepared nanoherbicides integrated with multi-stimuli responses to amylase, competitors at leaf interface, and elevated temperature under sunlight to control the PQ release for efficient weed control. The as-prepared nanoherbicides appeared low cytotoxicity to human normal cells *in vitro* and high mouse survival rate *in vivo*, because the coating of CDP gatekeepers and the robust π–π effects between PQ (electron deficiency) and MCN (electron enrichment) could availably avoid the release of trapped PQ under the simulated human gastric or intestinal conditions, which indicated it is safe for users from the root against the challenge of gastrointestinal absorption of PQ. Even through the nanoherbicides inevitably contact with water or intake by beneficial insects, they appear good biosafety on zebrafish and honeybees. It is clearly shown the nanoherbicides have high herbicidal efficacy and low risks to non-target species, and could promote the open use of PQ in agriculture.

## Compliance with Ethics Requirements

All animal protocols were approved by the Institutional Animal Care and Use Committee of Nanjing University (License: SYXK(Su) 2019–0056).

## Declaration of competing interest

The authors declare that they have no known competing financial interests or personal relationships that could have appeared to influence the work reported in this paper.
