# Supplementary material for: Bio-friendly multi-stimuli responsive α-CD polymer-gated mesoporous carbon nanoherbicides for enhanced paraquat delivery
Source: J Adv Res. 2024 Dec 12;76:1–16. doi: 10.1016/j.jare.2024.12.005 (PMC12793760; doi:10.1016/j.jare.2024.12.005)
Supplement: Supplementary Data 1 [file mmc1.docx]

**Bio-friendly Multi-stimuli Responsive α-CD Polymer-Gated Mesoporous Carbon Nanoherbicides for Enhanced Paraquat Delivery**

Jiangtao Dong,^a,b,*^ Guoquan Wang,^a^ Xiaona Li,^d^ Anhui Han,^a^ Wanpeng Zhang,^a^ Yuhang Yue,^a^ Yue Yang,^a^ Yishan Wang,^a^ Bowen Yuan,^a^ Jiahui Wang,^a^ Yuhui Peng,^a^ Runqiang Liu,^a,b,*^ Si Chen,^c*^ and Xuezhong Du^d,*^

**EXPERIMENTAL SECTION**

**Materials**

Octadecyltrichlorosilane (OTS, ≥90%) and Pluronic F127 (PEO_106_PPO_70_PEO_106_, MW = 12600 Da) were from Sigma-Aldrich. Epichlorohydrin (EP), formaldehyde (37~40%), acetone, ethanol (EtOH), decanoic acid (DA, ≥ 98.5%), acetic acid (99.8%, GR), phenol, NaOH, acetonitrile, Tween-80, Na_2_SO_4_, H_2_SO_4_, HNO_3_, dimethylsulfoxide (DMSO), and dichloromethane were from Sinopharm Chemical Reagent Co., Ltd. (China). Paraquat (PQ, 98%) was obtained from RHAWN. Dicyclohexylcarbodiimide (DCC, 99%), N-hydroxysuccinimide (NHS, 98%), α−cyclodextrin (α−CD, 98%), ursolic acid (UA, 98%), and NH_2_-PEG-COOH (MW = 2000 Da) were from Aladdin. Bis(2-ethylhexyl) sulfosuccinate sodium salt (AOT, 96%) was from Shanghai Titan. Fluorescein5(6)-isothiocyanate (FITC, 96%) and α−amylase (50 U mg^–1^, BR grade) were from Shanghai Macklin. The zebrafish (*D. rerio*) and honeybees (*Apis mellifera L.*) were offered by Henan Institute of Science and Technology.

**Synthesis of MCN**

Small-size MCN was synthesized via the low-concentration hydrothermal method [S1]. In detail, Phenol (0.6 g), 37% formaldehyde (2.1 mL) aqueous solution, and 0.1 M NaOH (15 mL) aqueous NaOH solution were mixed and stirred at 70^o^C for 0.5 h to obtain low-molecular-weight phenolic resols, then Pluronic F127 (0.96 g) dissolved in 15 mL of water was added. The mixed solution was stirred at 69^o^C for about 3 h, then 50 mL of water was added to dilute the mixed solution. During the reaction, the color of the solution turned from colorless transparent to pink and then to crimson. After about 17 h, the reaction was terminated when a precipitate was appeared. During the reaction termination, the precipitate dissolved and disappeared completely, then 17.7 mL of solution was placed in stainless steel reactor, and followed by the addition of 56 mL of water, heating at 130^o^C for 24 h. The product was collected, and washed by water for 4–6 times, and dried naturally. The carbonization of the obtained product was administered at 700^o^C in an N_2_ atmosphere for about 3 h, and synchronously the Pluronic F127 templates were cleared in this step, thus the initial MCN was synthesized.

**Carboxylation of MCN**

The as-synthesized MCN was placed into the mixed acid solution consist of concentrated H_2_SO_4_ and HNO_3_ (v/v, 3:1) for carboxylation for about 4 h via ultrasound. Then, the carboxylated MCN was collected and washed by water for 6–8 times until the supernatant became neutral.

**Synthesis of PEG-Functionalized MCN**

The PEG stalks functionalized MCN was synthesized via the amidation reaction. One hundred milligrams of MCN was dispersed in 20 mL of anhydrous dichloromethane solution, EDC (0.16 g, 0.8 mmol) and NHS (0.10 g, 0.8 mmol) were added to activate the carboxyl group, and 20 μL of triethylamine as catalyst was added and reacted at room temperature for 12 h. NH_2_-PEG-COOH (100 mg, MW = 2000 Da) dissolved in DMSO was added to the solution. The reaction was continued for 24 h under N_2_ atmosphere in the dark. The product was collected by centrifugation and washed by water 3−4 times, dialyzed in water 3−4 times with a dialysis bag (MW cutoff = 7000), each time for 24 h. The PEG functionalized MCN (MCN-PEG) was obtained via vacuum drying at 60^o^C.

**Synthesis of α−CD Polymer (CDP) Gatekeepers**

The synthesis of water-soluble α−CD polymer (CDP) gatekeepers through the cross-linking of α−CD units and epichlorohydrin (EP) in basic solution was administered based on the modified method in the paper (Figure S1) [S2]. 5 g of α−CD (0.44 mmol) was dissolved in 8 mL of 30% NaOH aqueous solution, and stirred for 12 h at room temperature. Then the solution was heated to 30 ^o^C, and 1.723 mL of EP was added for polymerization. After 24 h, acetone was added to stop the reaction, and then acetone was removed via reduced pressure way. 6 M HCl was used to neutralize the solution. Then, the solution was evaporated via reduced pressure way, and precipitated by EtOH to obtain the final products. The ^1^H NMR, ^13^C NMR, and FTIR spectra of CDP are afforded in Figure S2–S4, respectively.

**Preparation of Nanoherbicides (PQ@MCN-PEG@CDP)**

MCN-PEG (40 mg) was dispersed in 6 mM technical PQ aqueous solution, and stirred for 24 h in the dark, the PQ loaded MCN-PEG were retrieved via centrifugation, and placed in 10 mL of 10 mg mL^–1^ CDP aqueous solution, and stirred for 24 h. The products were collected and washed by water 3−4 times to remove the superfluous CDP. The PQ@MCN-PEG@CDP nanoherbicides were obtained by freeze-dried way. The MCN-PEG@CDP counterparts without the technical PQ loading were obtained in the same procedure without the addition of the technical PQ.

**Controlled Release of the Technical PQ From Nanoherbicides**

The as-prepared PQ@MCN-PEG@CDP nanoherbicides of equal weight were added to various aqueous solution with 0.1% Tween-80. The aqueous solution at various pH (pH 9.0, 7.2, 5.0, 3.0, and 2.0), at different temperatures (25, 40, and 50 ^o^C), with 2% Na_2_SO_4_, UA and DA at various concentrations at pH 7.2, in presence of Tween-80 and AOT at different fractions of critical micelle concentration (CMC), and in presence of α−amylase were used for the controlled release of the technical PQ. A fraction of the target aqueous solution was extracted at the given time for the test of the released PQ via HPLC, and later the fresh aqueous media of equal volume was added at the same time.

**Wettability of MCN, MCN-PEG, and MCN-MEG@CDP**

The polished glass slides were decorated with octadecyltrichlorosilane (OTS) in absolute toluene, and then blow dry with nitrogen. The wettability of aqueous droplets with MCN, MCN-PEG, and MCN-PEG@CDP was test on the surface of OTS-decorated glass slides, respectively. MCN, MCN-PEG, and MCN-PEG@CDP were dispersed in aqueous solutions (200 μg mL^–1^ of the final concentration) with the additives (Tween-80 or AOT with different fractions of CMC), respectively. The contact angle of each sample (5 μL) was measured on the surface of hydrophobic glass slide.

**Deposition and Retention of PQ@MCN-PEG@CDP**

The aqueous dispersion of PQ@MCN-PEG@CDP or FITC@MCN-PEG@CDP (0.5 mg mL^–1^, 10 mL) was sprayed uniformly on the surface of weed leaves at a 60° bevel to the horizontal, respectively. To mimic the effect of rain washing, 15 mL of water was sprayed on weed leaf surface after the aqueous droplets on the leaf surface had naturally dried at room temperature. Then, the treated weed leaves (before and after washing) were observed by SEM or confocal laser scanning microscope. The excitation wavelength of FITC was 488 nm. Each experiment was repeated three times.

**The** **Safety Evaluation of PQ@MCN-PEG@CDP *in Vitro*** ***/ in vivo***

The human normal hepatic cells (LO-2) were used to test the cytotoxicity of MCN-PEG@CDP and PQ@MCN-PEG@CDP. The fresh culture media containing MCN-PEG@CDP and PQ@MCN-PEG@CDP with LO-2 cells were co-incubated for 24 and 48 h, respectively. The cell viability of LO-2 cells was determined using MTT assay.

Female C57BL/6 mice (6 weeks old) were provided by Animal Experiment Center of School of Life Sciences of Nanjing University and housed with a 12 h light/dark cycle at 25^o^C, 40% relative humidity, and food and water ad libitum. All animal studies were performed in accordance with Animal Research: Reporting In Vivo Experiments (ARRIVE) guidelines. All animal protocols were approved by the Institutional Animal Care and Use Committee of Nanjing University (License: SYXK(Su) 2019-0056).

Female C57BL/6 mice (6 weeks old) were intragastrically administrated with pure active ingredient PQ at the dosage of 20 mg kg^-1^, MCN-PEG@CDP at 290 mg kg^-1^, and PQ@MCN-PEG@CDP at 290 mg kg^-1^ (equivalent to the PQ dose of 7 mg kg^-1^) on the first day, respectively, and their survival rates were examined over a period of 14 days (*n* = 6). Meanwhile, the body weights of the mice in different groups were measured. At the end of experiment, all mice were sacrificed, and the blood were collected for testing hepatic damage biomarkers (ALT and AST) and renal function biomarkers (UA and BUN). The heart, liver, spleen, lung, and kidney were collected for performing H&E staining according to the standard method.

**Herbicidal Efficacy of PQ@MCN-PEG@CDP Nanoherbicides**

The technical PQ (0.2 mg mL^–1^), MCN-PEG@CDP (3.0 mg mL^–1^), and PQ@MCN-PEG@CDP (1.0 and 3.0 mg mL^–1^, respectively same as 0.068 and 0.2 mg mL^–1^ of the technical PQ) were dispersed in aqueous solutions with 0.1% Tween-80, respectively. Each sample (*n* = 3) was sprayed against outdoor weeds (*Cynodon dactylon*) in an area of 0.16 m^2^ (0.4 m × 0.4 m). Eight milliliter of each sample was sprayed for the first three days. The damage levels of weeds (control efficacy against weeds herein) were classified as the following level: level 0, the whole weeds normal; level 1, discoloration of weed leaves or roots; level 2, ≥ 1/4 of the whole weeds withered and yellowed; level 3, ≥ 1/2 of the whole weeds withered and yellowed; level 4, ≥ 3/4 of the whole weeds withered and yellowed; level 5, the whole weeds withered to death. Damage rate (%) = Σ(Number of damaged weeds at various levels × Relative level)/(Total number of weeds × 5) × 100. Each experiment was repeated three times.

**Safety of PQ@MCN-PEG@CDP Nanoherbicedes on Non-Target Species Zebrafish and Honeybees**

In order to evaluate the effects of PQ@MCN-PEG@CDP nanoherbicides on aquatic ecosystem, zebrafish (*D. rerio*) were used as experimental species. The semi-static method was used to evaluate the acute toxicity of zebrafish. They were acclimated under experimental conditions for 7 days before the experiment. zebrafish were randomly selected with a total length of 1.5 ~ 2.5cm, normal body color, healthy and lively, and an average body weight of 0.200 g. The experimental water was filtered aerated tap water, and the temperature of 25±1^o^C, pH adjusted to 6.5 by 1.0 mol/L HCl, hardness of 114 mg/L, light/dark ratio of 14/10 h. Stop feeding 24 h before formal trial. The volume of the test solution was 500 mL. A series of concentrations of PQ@MCN-PEG@CDP nanoherbicides (respectively same as 25, 50, 75, 100, and 125 μg mL^–1^ of the technical PQ) were set up in the experiment with 10 zebrafish per group (*n* = 8 × 3). The test solution was changed at 24, 48, 72, and 96 h after the start of the test, and pH, water temperature and dissolved oxygen content were measured. The poisoning symptoms and death of zebrafish were observed and recorded at each designed point. The criteria for determining death is to touch the tail of the fish with a glass rod and remove the dead fish in time. Each experiment was repeated three times.

Honeybees (*Apis mellifera L*.) were artificially raised in climate incubator (26 ± 1^o^C, 60% humidity) with 50% sucrose solution. The honeybees were administrated for a 24-hour adaptation period in climate incubator. Afterward, observe and eliminate dead or abnormal individual. The ingestion method was used to evaluate the acute oral toxicity of honeybees. Two hours of starvation treatment before the experiment. Fifty honeybees (3 parallel trials for each group) per group were randomly selected and incubated in a feeding box, and fed with 50% sucrose solution containing the usages of 1470 and 2945 μg mL^–1^ PQ@MCN-PEG@CDP nanoherbicides (respectively same as 100 and 200 μg mL^–1^ of the technical PQ). Record the numbers of deaths after 24 and 48 h respectively, and calculate lethal rate of honeybees.

**Data Analysis.**

All treatments were run with at least 3 replicates. The median effective concentration (*EC*_50_) for the survival curves was calculated by probit regression analysis. Comparisons of the leaf retention amount, fluorescence image intensity, cell viability, liver and renal function biomarkers, herbicidal efficacy, survival rates, and biosecurity results were analyzed using one-way analysis of variance (ANOVA) and Duncan’s multiple range test. ^*^*P* < 0.05 and ^**^*P* < 0.01 were considered as significant and highly significant, respectively. The statistical analyses were performed using Data Processing Station SPSS 22.0 (IBM, USA). The charts and graphs of data were presented by OriginPro 8.5 (OriginLab, USA). Graphed data are shown as means ± standard errors.

**Figure S1.** Synthetic route to CDP.


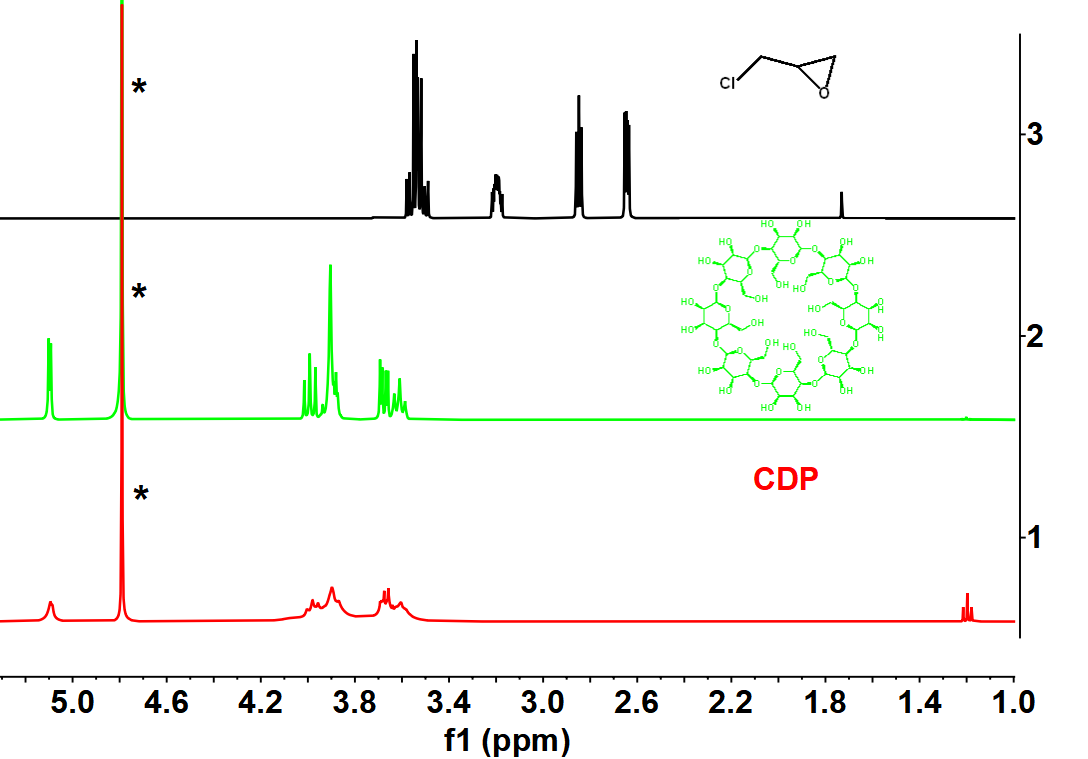


**Figure S2.** ^1^H NMR spectrum (400 MHz) of epichlorohydrin (EP), β-CD, and CDP in D_2_O.


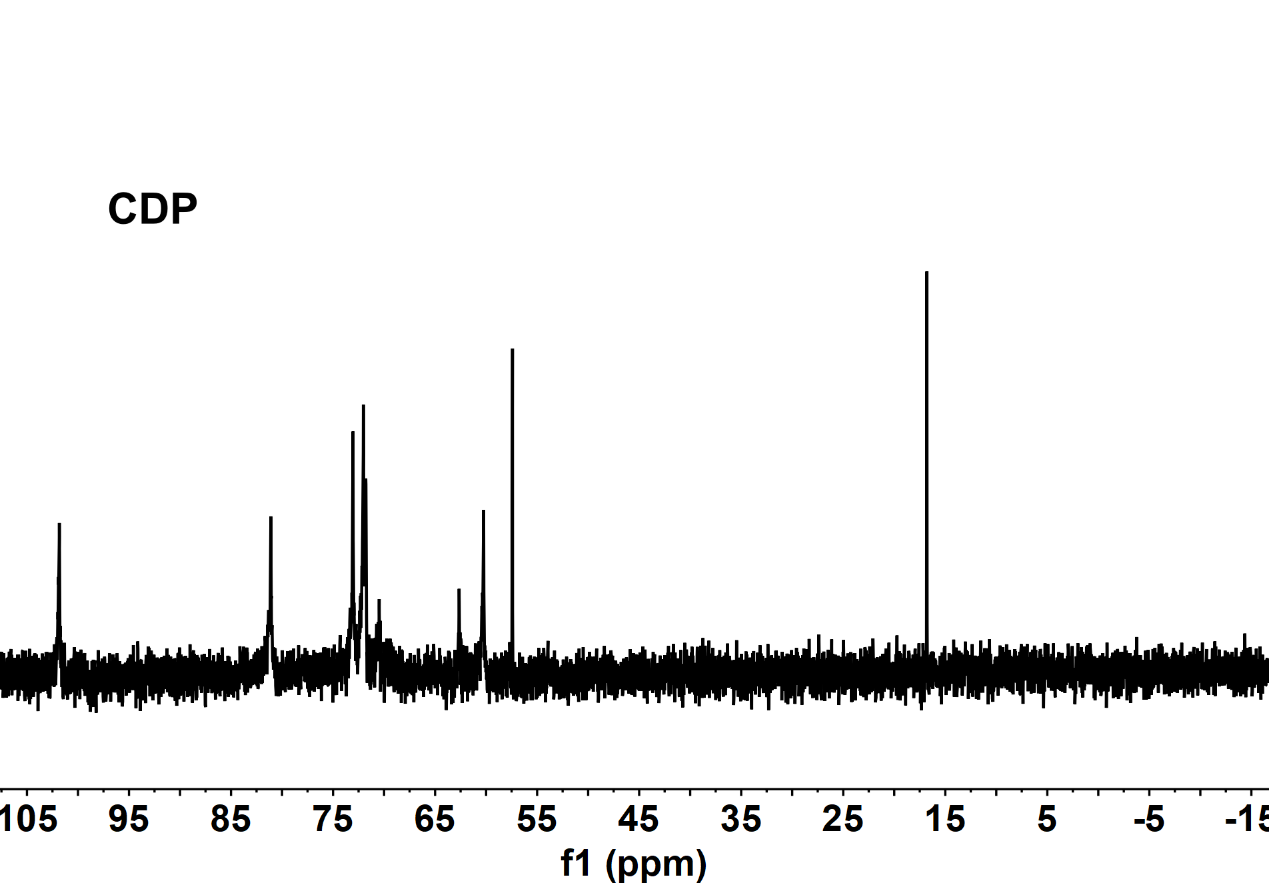


**Figure S3.** ^13^C NMR spectrum (400 MHz) of CDP in D_2_O.





**Figure S4.** FTIR spectra of epichlorohydrin (EP), β-CD, and CDP.

For the as-synthesized CDP, a broad band around 3321 cm^−1^ was assigned to the O−H stretching vibration, the weak peak at 2874 cm^−1^ was to the antisymmetric CH_2_ stretching vibration, the peaks in the range of 1430−1265 cm^−1^ were to the C−O−H bending vibrations of primary and secondary alcohols [S3]. The band at 1148 cm^−1^ was attributed to the out-of-phase C−C−O stretching vibrations of secondary alcohols and the antisymmetric C−O−C stretching vibrations [S3]. A series of strong bands at 1070 and 1021 cm^−1^ were assigned to the out-of-phase C−C−O stretching vibrations of primary and secondary alcohols [S3]. The band at 846 cm^−1^ was owing to the in-phase C−C−O stretching vibrations of primary and secondary alcohols and the symmetric C−O−C stretching vibrations [S3].





**Figure S5.** Raman spectrum of MCN





**Figure S6.** Standard curve of PQ determined by HPLC





**Figure S7.** Cell viability of technical PQ (7 μg mL^-1^) for incubation 24 and 48 h with human LO-2 cells (*n* = 6). The data are the mean values ± standard errors.





**Figure S8.** Contact angles of the aqueous droplets of MCN-PEG@CDP and related NPs on the surfaces of hydrophobic slides in the existence of Tween-80 or AOT at various fractions of CMC (*n* = 3). The data are the mean values ± standard errors.





**Figure S9.** Damage rate and damage level of the PQ@MCN-PEG@CDP nanoherbicides at various doses after spraying against outdoor weeds (*Cynodon dactylon*) compared with the technical PQ (0.2 mg mL^–1^) under sunlight over time (*n* = 3). The data are the mean values ± standard errors.


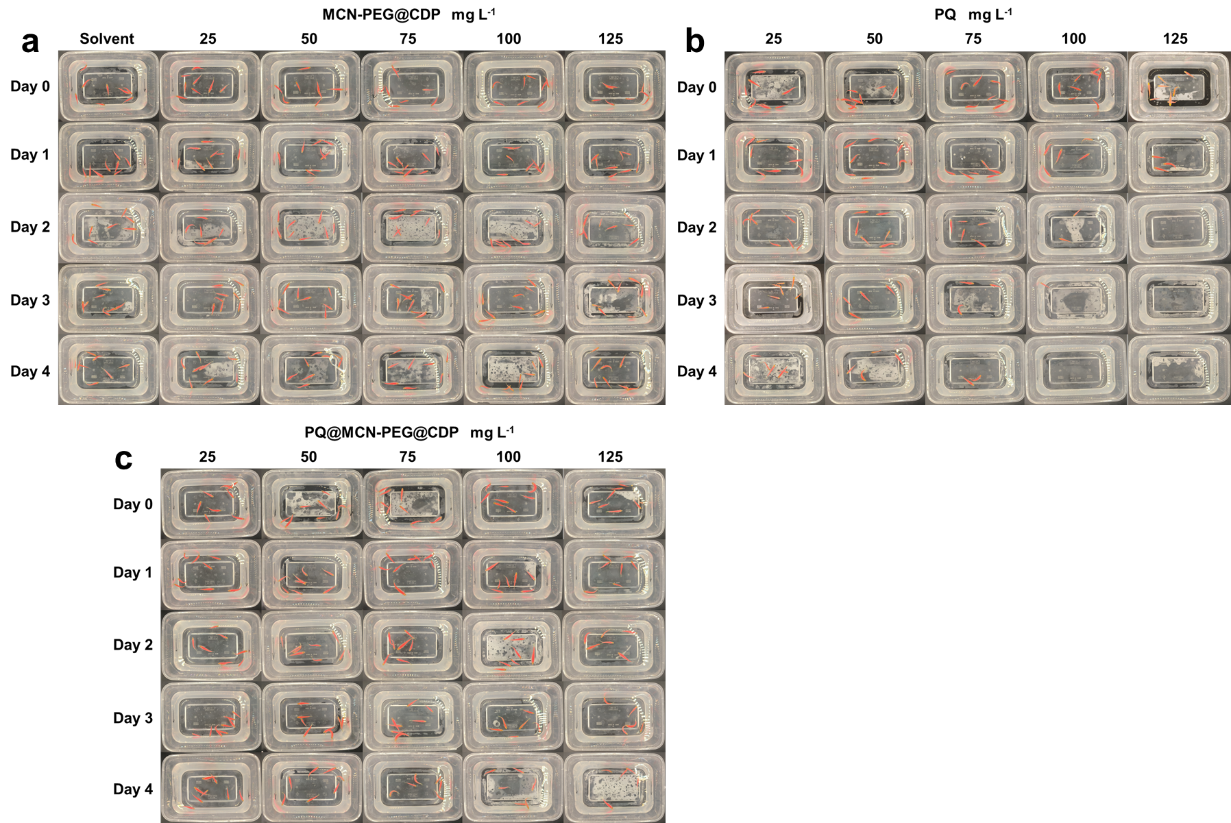


**Figure S10.** The photograph of (a) MCN-PEG@CDP, (b) technical PQ and (c) PQ@MCN-PEG@CDP at different PQ dosages (same as 25, 50, 75, 100, and 125 mg L^–1^ of technical PQ) against zebrafish with different incubation time (*n* = 8 × 3).

**Table S1.** Hydrodynamic sizes and zeta potentials of MCN, MCN-PEG, and MCN-MEG@CDP NPs.

| sample | hydrodynamic size [nm] | PDI | zeta potential [mV] |
| --- | --- | --- | --- |
| MCN | 92 | 0.137 | –39.4 |
| MCN-PEG | 105 | 0.095 | –36.6 |
| MCN-PEG@CDP | 119 | 0.033 | –47.7 |

**Table S2.** BET specific surface areas, pore volumes, and pore diameters of MCN relevant NPs calculated by the nitrogen adsorption–desorption isotherms.

| sample | BET surface area [cm^2^ g^–1^] | pore volume [cm^3^ g^–1^] | pore diameter [nm] |
| --- | --- | --- | --- |
| MCN | 928 | 0.42 | 1.7/2.1/2.5 |
| MCN-PEG | 876 | 0.34 | 1.7/2.1/2.5 |
| PQ@MCN-PEG | 230 | 0.17 | 1.7/2.1 |
| PQ@MCN-PEG@CDP | 41 | 0.06 | – |

**Table S3** Hydrodynamic sizes of PQ@MCN-PEG@CDP nanoherbicides in aqueous solution with 0.1% Tween-80 on the different days (*n* = 3). The data are the mean values ± standard errors.

| day | hydrodynamic size [nm] | PDI |
| --- | --- | --- |
| 1 | 131±7.1 | 0.085 |
| 3 | 136±5.4 | 0.076 |
| 6 | 129±4.3 | 0.107 |
| 9 | 134±5.2 | 0.114 |
| 12 | 127±6.1 | 0.116 |
| 15 | 140±4.9 | 0.106 |

**Table S4** Contact angles of the aqueous droplets on the surfaces of hydrophobic galss slides in the presence of Tween-80 at various critical micelle concentrations (*n* = 3) (CMC_Tween-80_ = 0.014 g L^–1^): (I) without NPs; (II) containing MCN; (III) containing MCN-PEG, (IV) containing MCN-PEG@CDP. The data are the mean values ± standard errors.

| Sample | contact angle [°] | | | | | | |
| --- | --- | --- | --- | --- | --- | --- | --- |
|  | Water | Tween-80  [0.05 CMC] | Tween-80  [0.1 CMC] | Tween-80  [0.5 CMC] | Tween-80  [1.0 CMC] | Tween-80  [3.0 CMC] | Tween-80  [5.0 CMC] |
| blank | 120.7±6.4 | 104.1±5.1 | 99.1±4.6 | 93.2±3.7 | 80.6±3.6 | 74.0±3.8 | 70.4±4.2 |
| MCN | 113.9±4.9 | 97.0±5.0 | 88.3±4.1 | 79.8±3.7 | 76.2±3.4 | 63.9±2.8 | 59.7±3.5 |
| MCN-PEG | 100.3±4.2 | 92.5±3.9 | 81.2±3.8 | 74.9±4.2 | 70.3±4.1 | 60.1±3.8 | 55.6±3.1 |
| MCN-PEG@CDP | 95.8±4.2 | 88.1±3.1 | 76.9±2.7 | 71.0±3.1 | 65.2±2.9 | 55.9±2.4 | 51.0±3.1 |

**Table S5** Contact angles of the aqueous droplets on the surfaces of hydrophobic galss slides in the presence of AOT at various critical micelle concentrations (*n* = 3) (CMC_AOT_ = 2.5 mM): (I) without NPs; (II) containing MCN; (III) containing MCN-PEG, (IV) containing MCN-PEG@CDP. The data are the mean values ± standard errors.

| Sample | contact angle [°] | | | | | | |
| --- | --- | --- | --- | --- | --- | --- | --- |
|  | Water | AOT  [0.05 CMC] | AOT  [0.1 CMC] | AOT  [0.5 CMC] | AOT  [1.0 CMC] | AOT  [3.0 CMC] | AOT  [5.0 CMC] |
| blank | 120.7 ± 6.4 | 95.6 ± 4.7 | 77.9 ± 4.2 | 55.7 ± 3.7 | 44.6 ± 3.2 | 32.3 ± 2.7 | 22.5 ± 2.1 |
| MCN | 113.9 ± 4.9 | 95.0 ± 3.3 | 79.8 ± 3.4 | 62.1 ± 4.6 | 53.7 ± 3.1 | 35.7 ± 2.9 | 26.1 ± 2.3 |
| MCN-PEG | 100.3 ± 4.2 | 84.4 ± 3.7 | 72.9 ± 4.1 | 59.3 ± 2.1 | 48.0 ± 2.6 | 33.7 ± 2.2 | 25.0 ± 1.9 |
| MCN-PEG@CDP | 95.8 ± 4.2 | 75.6 ± 3.5 | 64.5 ± 2.7 | 53.6 ± 3.1 | 41.4 ± 2.8 | 28.5 ± 2.6 | 22.0 ± 1.7 |

**Table S6** The leaf retention amount of PQ@MCN-PEG@CDP (converted into amount of technical PQ according to the loading rate of PQ) on weed (*Cynodon dactylon*) leaf surfaces before and after washing with water (mg cm^-2^). The data are the mean values ± standard errors. Comparisons of the cell viability were analyzed using one-way analysis of variance (ANOVA) and Duncan’s multiple range test (*n* = 3, *P* < 0.05, *P* < 0.01).

| Sample | Before  washing | After  washing | Retention rate of  free PQ / % | significance of difference *^a^* | |
| --- | --- | --- | --- | --- | --- |
|  |  |  |  | 0.05 | 0.01 |
| PQ@MCN-PEG@CDP | 0.185±0.097 | 0.158±0.091 | 85.446±9.077 | *P<*0.05 | *P<*0.01 |
| Technical PQ | 0.187±0.016 | 0.048±0.013 | 25.623±1.253 | - | - |

^a^ according to a least significant difference test compared with technical PQ.

**Table S7** The fluorescence image intensity of free FITC and FITC@MCN-PEG@CDP on weed (*Cynodon dactylon*) leaf surfaces before and after washing with water. The data are the mean values ± standard errors. Comparisons of the cell viability were analyzed using one-way analysis of variance (ANOVA) and Duncan’s multiple range test (*n* = 3, *P* < 0.05, *P* < 0.01).

| Sample | Before  washing | After  washing | Retention rate of  free FITC / % | significance of difference *^a^* | |
| --- | --- | --- | --- | --- | --- |
|  |  |  |  | 0.05 | 0.01 |
| FITC@MCN-PEG@CDP | 76.81±1.53 | 70.84±2.31 | 92.24±1.80 | *P<*0.05 | *P<*0.01 |
| Free FITC | 64.63±1.99 | 21.50±3.36 | 33.26±3.29 | - | - |

^a^ according to a least significant difference test compared with free FITC.

**Table S8** Cell viability of MCN-PEG@CDP at 100 μg mL^-1^, PQ@MCN-PEG@CDP at 100 μg mL^-1^ (same as 7 μg mL^-1^ of the technical PQ), and technical PQ at 7 μg mL^-1^ for cultivation 24 and 48 h with human LO-2 cells. The data are the mean values ± standard errors. Comparisons of the cell viability were analyzed using one-way analysis of variance (ANOVA) and Duncan’s multiple range test (*n* = 6, *P* < 0.05, *P* < 0.01).

| Sample | Time / h | Cell viability / % | significance of difference *^a^* | |
| --- | --- | --- | --- | --- |
|  |  |  | 0.05 | 0.01 |
| MCN-PEG@CDP | 24 | 85.97±8.13 | *P<*0.01 | *P<*0.01 |
|  | 48 | 84.88±8.04 | *P<*0.01 | *P<*0.01 |
| PQ@MCN-PEG@CDP | 24 | 83.59±7.52 | *P<*0.01 | *P<*0.01 |
|  | 48 | 76.69±7.64 | *P<*0.01 | *P<*0.01 |
| Technical PQ | 24 | 47.43±2.72 | ***-*** | ***-*** |
|  | 48 | 31.25±3.51 | - | - |

^a^ according to a least significant difference test compared with technical PQ.

**Table S9** Herbicidal efficacy of the PQ@MCN-PEG@CDP nanoherbicides at different concentrations (1.0 and 3.0 mg mL^–1^, corresponding to PQ at 0.068 and 0.2 mg mL^–1^) sprayed against outdoor weed (*cynodon dactylon*) under natural sunlight with time, together with solvent (0.1% Tween-80), MCN-PEG@CDP (3.0 mg mL^–1^), PQ (0.2 mg mL^–1^) for comparison. The data are the mean values ± standard errors. Comparisons of the cell viability were analyzed using one-way analysis of variance (ANOVA) and Duncan’s multiple range test (*n* = 3, *P* < 0.05, *P* < 0.01).

| group | dosage /  mg mL^–1^ | day | damage level | damage rate /  % | significance of difference *^a^* |
| --- | --- | --- | --- | --- | --- |
| solvent | / | 0 | 0 | 0 | / |
|  |  | 1 | 0 | 0 | / |
|  |  | 2 | 0 | 0 | / |
|  |  | 3 | 0 | 0 | / |
|  |  | 4 | 0 | 0 | / |
|  |  | 6 | 0 | 0 | / |
|  |  | 9 | 0 | 0 | / |
| PQ | 0.2 | 0 | 0 | 0 | / |
|  |  | 1 | 3 | 58.46±8.1 | *P<*0.01 |
|  |  | 2 | 3 | 64.66±5.3 | *P<*0.01 |
|  |  | 3 | 3 | 70.14±6.2 | *P<*0.01 |
|  |  | 4 | 4 | 82.94±4.9 | *P<*0.01 |
|  |  | 6 | 5 | 94.16±6.4 | *P<*0.01 |
|  |  | 9 | 5 | 98.55±2.3 | *P<*0.01 |
| MCN-PEG@CDP | 3.0 | 0 | 0 | 0 | / |
|  |  | 1 | 0 | 0 | / |
|  |  | 2 | 0 | 0 | / |
|  |  | 3 | 0 | 0 | / |
|  |  | 4 | 0 | 0 | / |
|  |  | 6 | 0 | 0 | / |
|  |  | 9 | 0 | 0 | / |
| PQ@MCN-PEG@CDP | 1.0 | 0 | 0 | 0 | / |
|  |  | 1 | 1 | 5.35±2.9 | *P<*0.05 |
|  |  | 2 | 1 | 8.67±4.3 | *P<*0.05 |
|  |  | 3 | 2 | 26.18±3.9 | *P<*0.01 |
|  |  | 4 | 3 | 59.61±7.4 | *P<*0.01 |
|  |  | 6 | 4 | 86.46±6.8 | *P<*0.01 |
|  |  | 9 | 5 | 94.55±5.3 | *P<*0.01 |
|  | 3.0 | 0 | 0 | 0 | / |
|  |  | 1 | 2 | 25.08±3.7 | *P<*0.01 |
|  |  | 2 | 2 | 31.33±5.4 | *P<*0.01 |
|  |  | 3 | 3 | 75.47±7.3 | *P<*0.01 |
|  |  | 4 | 3 | 79.66±6.7 | *P<*0.01 |
|  |  | 6 | 4 | 86.93±6.2 | *P<*0.01 |
|  |  | 9 | 5 | 98.66±3.7 | *P<*0.01 |

^a^ according to a least significant difference test compared with solvent (0.1% Tween-80).

**Table S10** Survival rates of PQ@MCN-PEG@CDP, MCN-PEG@CDP, and Technical PQ at different PQ dosages (mg L^–1^) against zebrafish with different incubation time (*n* = 3). The data are the mean values ± standard errors.

| sample | time [day] | survival rate [%] | | | | |
| --- | --- | --- | --- | --- | --- | --- |
|  |  | 25 [mg L^–1^] | 50 [mg L^–1^] | 75 [mg L^–1^] | 100 [mg L^–1^] | 125 [mg L^–1^] |
| PQ@MCN-PEG@CDP | 1 | 100 | 100 | 100 | 87.50±4.06 | 87.50±3.79 |
|  | 2 | 100 | 100 | 87.50±2.67 | 87.50±3.09 | 75.00±1.85 |
|  | 3 | 100 | 100 | 87.50±1.76 | 75.00±1.37 | 62.50±2.49 |
|  | 4 | 100 | 87.5±3.72 | 75.00±2.64 | 75.00±1.93 | 62.50±3.88 |
| MCN-PEG@CDP | 1 | 100 | 100 | 100 | 100 | 100 |
|  | 2 | 100 | 100 | 100 | 100 | 100 |
|  | 3 | 100 | 100 | 100 | 100 | 100 |
|  | 4 | 100 | 100 | 100 | 100 | 100 |
| Technical PQ | 1 | 100 | 100 | 75.00±3.77 | 62.50±3.62 | 37.50±2.73 |
|  | 2 | 100 | 75.00±2.34 | 62.50±4.12 | 25.00±2.46 | 0 |
|  | 3 | 87.50±4.76 | 62.50±3.87 | 37.50±1.75 | 12.50±1.49 | 0 |
|  | 4 | 75.00±4.74 | 50.00±2.73 | 25.00±3.02 | 0 | 0 |

**Table S11** Biosecurity of PQ@MCN-PEG@CDP, MCN-PEG@CDP, and Technical PQ against zebrafish with different incubation time. The data are the mean values ± standard errors. Comparisons of the cell viability were analyzed using one-way analysis of variance (ANOVA) and Duncan’s multiple range test (*n* = 3, *P* < 0.05, *P* < 0.01).

| sample | time [day] | *EC*_50_ [mg L^–1^] | linear fitting equation | *R*^2^ | significance of difference *^a^* |
| --- | --- | --- | --- | --- | --- |
| CK | 1 | - | - | - | - |
|  | 2 | - | - | - | - |
|  | 3 | - | - | - | - |
|  | 4 | - | - | - | - |
| PQ@MCN-PEG@CDP | 1 | 230.575±10.12 | *-* | - | *P<*0.01 |
|  | 2 | 189.579±6.58 | *y* = 2.04*x* + 5.06 | 0.685 | *P<*0.01 |
|  | 3 | 140.641±8.16 | *y* = 3.75*x* + 8.18 | 1.000 | *P<*0.01 |
|  | 4 | 162.421±7.65 | *y* = 1.90*x* + 4.34 | 0.911 | *P<*0.01 |
| MCN-PEG@CDP | 1 | - | - | - | - |
|  | 2 | - | - | - | - |
|  | 3 | - | - | - | - |
|  | 4 | - | - | - | - |
| Technical PQ | 1 | 109.097±8.64 | *y* = 4.40*x* + 8.98 | 0.946 | *P<*0.01 |
|  | 2 | 73.154±4.34 | *y* = 4.32*x* + 8.13 | 0.873 | *P<*0.01 |
|  | 3 | 54.694±3.76 | *y* = 3.66*x* + 6.38 | 0.962 | *P<*0.01 |
|  | 4 | 42.584±4.65 | *y* = 2.76*x* + 4.58 | 0.978 | *P<*0.01 |

^a^ according to a least significant difference test compared with control blank (CK).

**Table S12.** Safety of PQ@MCN-PEG@CDP nanoherbicides to *Apis mellifera L*. at the doses of 1470 and 2945 μg mL^-1^ (equivalent to technical PQ doses of 100 and 200 μg mL^-1^, respectively) comparing with solvent control containing 0.1% Tween-80 (CK), PQ@MCN-PEG@CDP (2940 μg mL^-1^), and technical PQ (200 μg mL^-1^) with different incubation time. The data are the mean values ± standard errors. Comparisons of the cell viability were analyzed using one-way analysis of variance (ANOVA) and Duncan’s multiple range test (*n* = 50 × 3, *P* < 0.05, *P* < 0.01).

| sample | dose /  μg mL^–1^ | time [h] | number of deaths | lethal rate [%] | *significance of difference ^a^* |
| --- | --- | --- | --- | --- | --- |
| CK | / | 24 | 2.3±0.6 | 4.67±1.15 | / |
|  |  | 48 | 2.7±0.6 | 5.33±1.15 | / |
| technical PQ | 200 | 24 | 15.7±2.5 | 31.33±5.03 | *P<*0.01 |
|  |  | 48 | 32.0±2.6 | 64.00±5.29 | *P<*0.01 |
| MCN-PEG@CDP | 2945 | 24 | 3.3±0.6 | 6.67±1.15 | *P>*0.05 |
|  |  | 48 | 4.3±1.2 | 8.67±2.31 | *P>*0.05 |
| PQ@MCN-PEG@CDP | 1470 | 24 | 4.7±0.6 | 9.33±1.15 | *P>*0.05 |
|  |  | 48 | 7.7±0.6 | 15.33±1.15 | *P<*0.05 |
|  | 2945 | 24 | 8.7±1.5 | 17.33±3.06 | *P<*0.05 |
|  |  | 48 | 11.7±1.5 | 23.33±3.06 | *P<*0.01 |

^a^ according to a least significant difference test compared with control blank (CK).

**Reference**

[S1] Fang, Y.; Gu, D.; Zou, Y.; Wu, Z.; Li, F.; Che, R.; Deng, Y.; Tu, B.; Zhao, D. Low Concentration Hydrothermal Synthesis of Biocompatible Ordered Mesoporous Carbon Nanospheres with Tunable and Uniform Size, *Angew. Chem. Int. Ed*. **2010**, *49*, 7987–7991.

[S2] Renard, E.; Deratani, A.; Volet, G.; Sebille, B. Preparation and Characterization of Water Soluble High Molecular Weight β-Cyclodextrin-Epichlorohydrin Polymers. *Eur. Polym. J*. **1997**, *33*, 49–57.

[S3] Lin-Vien, D.; Colthup, N. B.; Fateley, W. G.; Grasselli, J. G.; *The Handbook of Infrared and Raman Characteristic Frequencies of Organic Molecules*, Academic Press, Inc., Harcourt Brace Jovanovich, Publishers, Boston, San Diego, New York, London, Sydney, Tokyo, Toronto, **1991**.
